# Degrading GPX4 via Biomimetic Nanoparticle‐Mediated In Situ Synthesis of Deep‐Learning‐Designed Binder‐Degron Chimeras for Prostate Cancer Therapy

**DOI:** 10.1002/advs.77932

**Published:** 2026-09-27

**Authors:** Si‐Han Zhang, Ye An, Na Zeng, Mei‐Cheng Liu, Jian‐Xuan Sun, Xi Gong, Jin‐Zhou Xu, Shao‐Gang Wang, Qi‐Dong Xia

**Affiliations:** ^1^ Department and Institute of Urology, Tongji Hospital, Tongji Medical College Huazhong University of Science and Technology Wuhan People's Republic of China; ^2^ Department of Urology, The First People's Hospital of Yunnan Province Kunming University of Science and Technology Affiliated Hospital Kunming People's Republic of China

**Keywords:** cancer research, chemistry, deep learning, degron, gpx4, intracellular, nanocarriers, prostate cancer, protein degradation, ubiquitin ligase

## Abstract

Glutathione peroxidase 4 (GPX4) is a central guardian against ferroptosis and a critical therapeutic target for castration‐resistant prostate cancer (CRPC). However, traditional GPX4 inhibitors are often limited by irreversible covalent binding and systemic toxicity. Here, we utilized the deep learning algorithm Bindcraft to design high‐affinity de novo binders targeting GPX4. To achieve GPX4 degradation, we developed a novel modality termed binder‐degron chimera (bdC), which integrates a de novo‐designed binder with a C‐terminal combinational degron. Mechanistically, bdC recruits the CUL2‐RING E3 ubiquitin ligase complex, leading to proteasome‐mediated GPX4 degradation. To circumvent the delivery barriers inherent to macromolecular therapeutics, the bdC was encoded into a plasmid vector and encapsulated within a biomimetic nanocarrier featuring a PSMA‐targeted and CD47‐camouflaged surface (pc‐CMNP). This platform enables the in situ synthesis of bdCs specifically within prostate cancer cells, driving robust GPX4 degradation and triggering potent ferroptosis. Our work establishes a versatile framework combining deep‐learning generative binders, combinational degrons, and biomimetic nanotechnology to degrade intracellular targets and provides a novel therapeutic modality for CRPC.

## Graphical abstract

A deep‐learning‐designed binder is fused to a combinatorial degron to create a binder‐degron chimera (bdC) that recruits the CUL2‐RING ubiquitin ligase complex. Encapsulation in a prostate‐cancer‐targeted, CD47‐camouflaged nanocarrier enables tumor‐specific in situ bdC expression, triggering GPX4 degradation and ferroptosis, thereby establishing a versatile platform for targeted protein degradation in castration‐resistant prostate cancer.

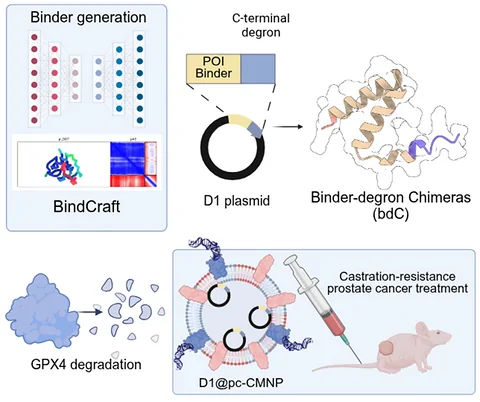

## 1 Introduction

Prostate cancer ranks as the second most frequently diagnosed malignancy among men worldwide [1]. Although androgen deprivation therapy (ADT) serves as the standard care, patients inevitably progress to castration‐resistant prostate cancer (CRPC), a stage characterized by poor prognosis and limited treatment options [2]. Emerging evidence demonstrated that inducing ferroptosis can effectively overcome resistance to androgen receptor (AR) signaling [3]. Therefore, developing novel therapeutic strategies that target ferroptosis pathways represents a promising avenue for the clinical management of CRPC.

Among the various regulators of the ferroptosis pathway, Glutathione peroxidase 4 (GPX4) functions as the central guardian against ferroptosis by neutralizing lipid peroxides [4]. However, GPX4 is traditionally considered a challenging target due to its relatively flat surface and the lack of deep binding pockets required for small‐molecule intervention. Although covalent inhibitors targeting the active site (Selenocysteine, Sec46), such as RSL3 and ML162, demonstrate potent activity in vitro, their clinical translation is hindered by poor pharmacokinetic profiles and potential off‐target toxicity [5]. Consequently, there is an urgent need to develop novel ligands, such as peptides, that can effectively target GPX4 with improved safety profiles. The rapid advancement of artificial intelligence (AI) and de novo protein design have facilitated the generation and screening of specific peptide binders for such challenging targets [6]. In this study, we utilized Bindcraft, a robust deep learning framework for protein design, in conjunction with HDOCK for peptide‐protein docking [7, 8]. Furthermore, Molecular Dynamics (MD) simulations were employed to validate stability and identify the most suitable binders for GPX4 inhibition [9].

Beyond simple inhibition, targeted Protein Degradation (TPD) has emerged as a revolutionary therapeutic modality to eliminate undruggable or resistant targets. The field was pioneered by proteolysis‐targeting chimeras (PROTACs), which hijack the ubiquitin‐proteasome system (UPS) to induce degradation [10]. Several small‐molecule PROTACs have already entered clinical trials, demonstrating remarkable efficacy [11]. Expanding on this, novel degraders targeting the autophagy‐lysosome pathway have been developed to degrade larger substrates or aggregates [12]. Traditional small‐molecule PROTACs often struggle to target proteins with ‘smooth’ surfaces that lack well‐defined binding pockets [13]. To address this, peptide‐based PROTACs were developed to leverage the broad recognition interface of peptides [14, 15]. Commonly, these peptide‐PROTACs are designed to recruit well‐established E3 ligases, such as Cereblon (CRBN), Mouse Double Minute 2 homolog (MDM2), or Von Hippel‐Lindau (VHL). But intriguingly, under physiological conditions, E3 ligases identify substrates through specific recognition signals known as “degrons”, which encompass diverse motifs such as the well‐characterized N‐degrons and C‐degrons located at protein termini [16, 17]. Utilizing these intrinsic motifs allows for the mimicry of natural degradation pathways [18]. Importantly, this strategy offers a unique benefit: certain degrons possess the capability to recruit multiple distinct E3 ligases, thereby broadening the scope and robustness of degradation [19].

**FIGURE 1:**
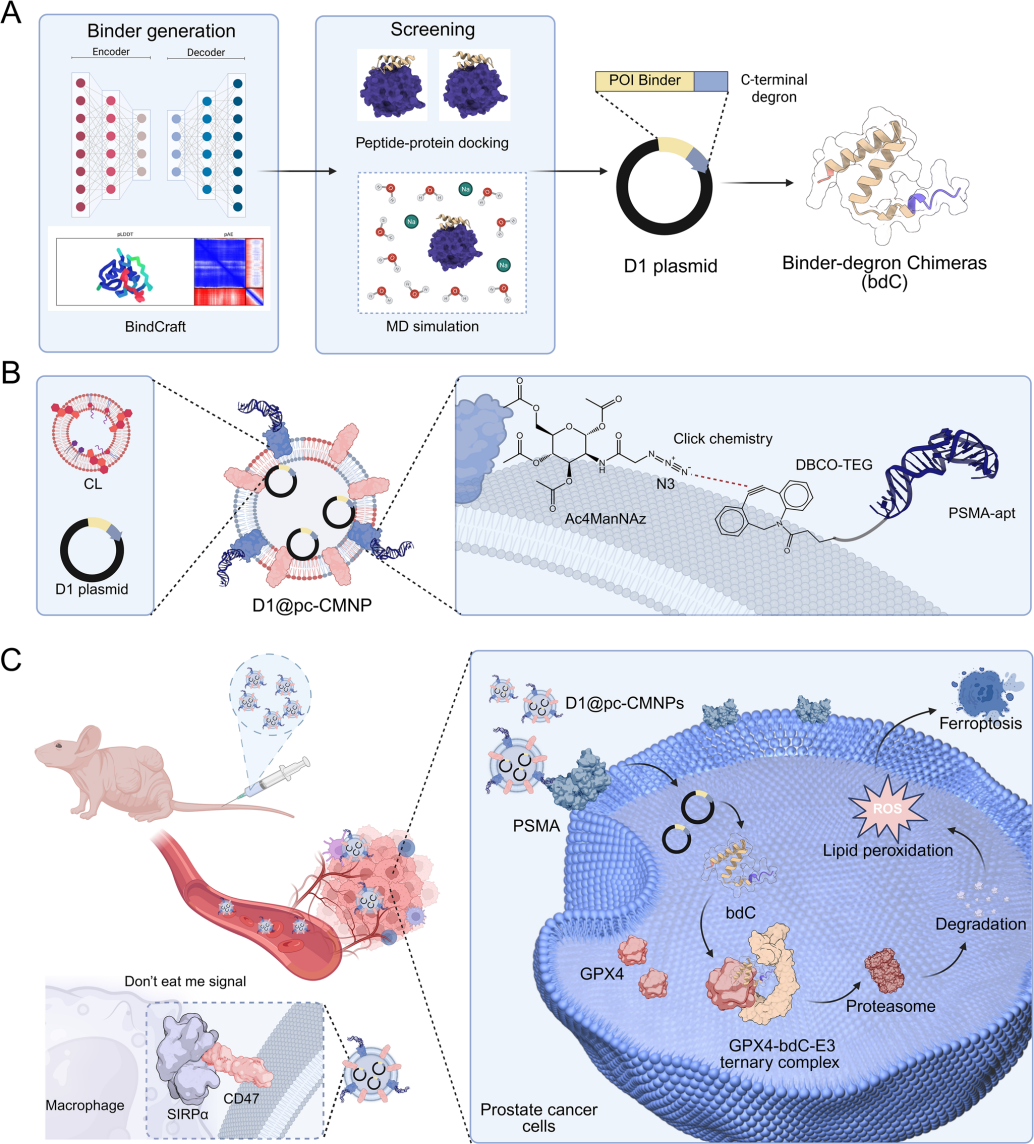
Illustration of D1@pc‐CMNP construction and its function in targeted GPX4 degradation in prostate cancer cells. (A) Design of the D1 plasmid expressing binder‐degron chimeras (bdC). (B) Construction of PSMA‐targeted, CD47‐expressing, cationic liposome (CL)‐incorporated cell membrane‐derived nanoparticles (pc‐CMNP) using click chemistry and membrane extrusion. (C) D1@pc‐CMNP evades macrophage phagocytosis via the “don't eat me” signal in vivo, targets PSMA‐expressing prostate cancer cells, and triggers ferroptosis through GPX4 degradation.

Despite the significant therapeutic potential of binder‐degron strategies, the clinical translation of these gene‐encoded modalities remains constrained by poor membrane permeability, enzymatic degradation, and limited bioavailability [20]. To overcome these barriers, bioPROTACs have emerged as a superior alternative. Similar to mRNA vaccine technology, which utilizes the host cell's own machinery to synthesize therapeutic proteins, bioPROTACs enable the intracellular expression of a target‐binding peptide fused to a functional E3 ligase domain [21]. This strategy facilitates autonomous protein production and precise targeted degradation, effectively overcoming the inherent limitations of exogenous peptide delivery [22, 23]. To enable the systemic delivery of such genetic payloads, nanoparticles (NPs) have been extensively developed as versatile drug delivery systems capable of enhancing tumor‐specific accumulation and minimizing off‐target toxicity. Crucially, the realization of such targeted delivery relies on precise surface engineering. Click chemistry has emerged as a powerful bioconjugation strategy in this regard, enabling the efficient and site‐specific attachment of targeting ligands onto the nanoparticle surface [24]. However, beyond targeting, a major hurdle is evading clearance by the mononuclear phagocyte system (MPS). Strategies hijacking the CD47‐SIRPα axis have proven particularly effective; functionalizing NPs to engage SIRPα on macrophages initiates a “don't eat me” signal, thereby inhibiting phagocytosis and prolonging circulation half‐life [25]. In this study, we integrated AI‐driven protein design with nanomedicine to develop a novel GPX4 degradation platform for CRPC therapy. Specifically, we utilized the deep learning algorithm Bindcraft to design a high‐affinity de novo binder for GPX4, which was subsequently engineered into a bdC capable of recruiting the CUL2‐RING E3 ubiquitin ligase complex. To achieve safe and efficient intracellular delivery, we developed a biomimetic nanocarrier (pc‐CMNP) that encapsulates bdC‐encoding plasmids. This system leverages click chemistry for precise PSMA‐targeting and incorporates a CD47‐camouflaged surface to evade immune clearance (Figure 1A–C). This integrated platform facilitates the in situ synthesis of bdCs specifically within prostate cancer cells, driving robust GPX4 degradation and triggering potent ferroptosis in prostate cancer.

## 2 Results

### 2.1 De Novo Design and Validation of Binders Targeting GPX4

**FIGURE 2:**
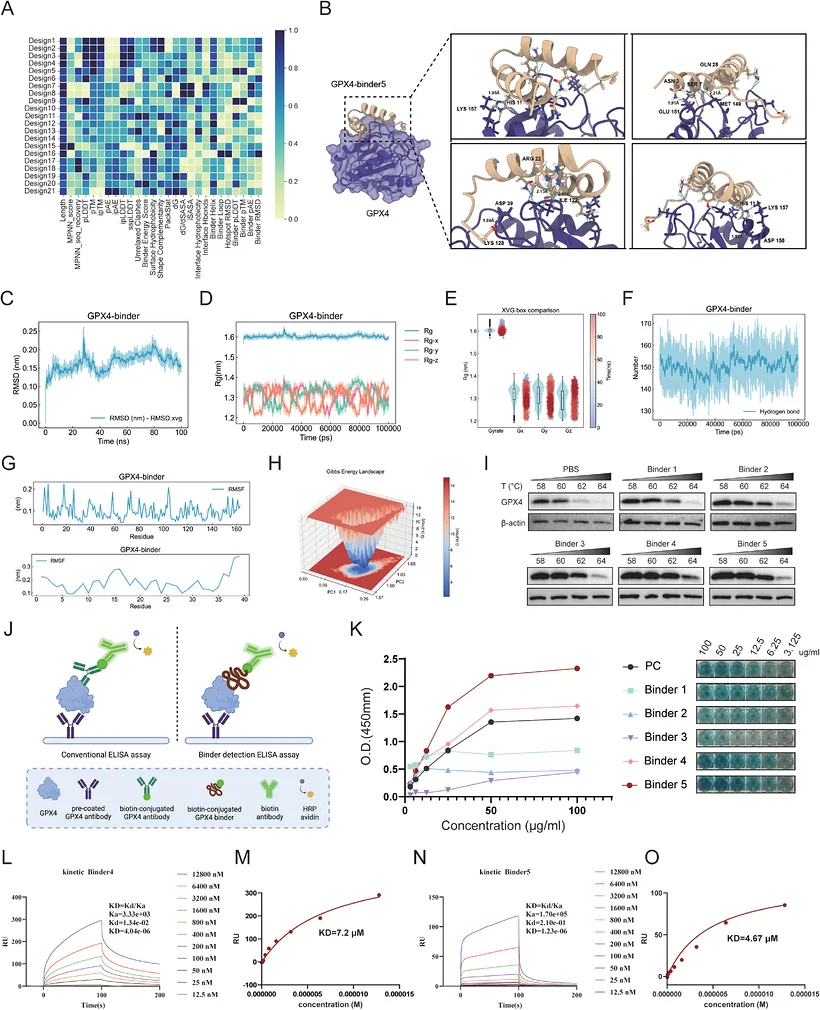
Generation, screening, and binding affinity verification of GPX4‐binders. (A) Features of the top 21 designs passing all filtration criteria in Bindcraft (each property normalized to the range of 0–1). (B) Schematic diagram of the docking between binder 5 and GPX4. Root‐Mean‐Square Deviation (RMSD, C), Radius of Gyration (Rg, D, E), number of hydrogen bonds (F), Root‐Mean‐Square Fluctuation (RMSF, G), and free energy landscape (H) from molecular dynamics simulations of the binder 5‐GPX4 complex. (I) Verification of the binding interaction of binders 1–5 with GPX4 using the cellular thermal shift assay (CETSA). (J) Schematic diagram of the binder detection ELISA assay. (K) Binding affinity of binders 1–5 to GPX4 at concentrations ranging from 3.125 µg/mL to 100 µg/mL (PC: positive control, GPX4 antibody). (L, N) Representative SPR sensorgrams showing real‐time association and dissociation kinetics of binder 4 (L) and binder 5 (N) across a concentration range of 12.5–12800 nM. (M, O) Steady‐state affinity fitting of binder 4 (M) and binder 5 (O) to GPX4, yielding an equilibrium dissociation constant (KD) of 7.2 µM.

By analyzing the crystal structure of GPX4, we identified several potential binding sites on its surface suitable for protein‐protein interaction using the cavity‐searching function in CB‐Dock2 (Table S1). To specifically target GPX4, a key regulator of ferroptosis, we employed Bindcraft to generate 157 distinct design trajectories, ultimately yielding 21 designs that satisfied all filtering criteria. Key properties of these designs, including length, pLDDT, pTM, PAE, binder energy score, and interface H‐bonds, were normalized to a range of 0–1 using Min‐Max scaling (Figure 2A). The top 10 binders ranked by Bindcraft (selecting only the highest‐ranked sequence among similar ones) were subjected to re‐docking and scoring evaluation using H‐DOCK (Figure S1). The results demonstrated that Binders 1, 5, and 8 possessed superior docking performance (Figure S2). Analysis of the i_pae, pTM, and Rg values for Binders 1–5 revealed that Binders 1, 4, and 5 exhibited lower final i_pae and Rg values, suggesting they maintained relatively stable binding with GPX4 throughout the design trajectories (Figure S3). Molecular dynamics (MD) simulations (100 ns) of the Binder 1–5/GPX4 complexes indicated that all five binders maintained low RMSD values and substantial, stable Solvent Accessible Surface Area (SASA) values, with Binder 5 consistently exhibiting the lowest Rg value (Figure S4). Root Mean Square Fluctuation (RMSF), indicative of residue flexibility, remained consistently below 4 Å for all five binders (Figure S5).

Docking analysis of Binder 5 with GPX4 revealed potential hydrogen bond formation at residues ASN3, SER7, HIS11, ARG22, GLN28, ASP39, ILE122, LYS128, MET149, LYS157, and ASP158 (Figure 2B). The GPX4‐Binder 5 complex system reached equilibrium after 50 ns, fluctuating around 1.5 Å (Figure 2C). The radius of gyration (Rg) of the complex in the x, y, and z directions fluctuated within the range of 1.2–1.4 nm (Figure 2D,E and Figure S7). The total number of hydrogen bonds in the complex varied between 140 and 160, stabilizing after 50 ns (Figure 2F). The RMSF values of the GPX4‐Binder 5 complex were predominantly below 2 Å, indicating low structural flexibility and high stability (Figure 2G). Furthermore, most amino acid residues in the GPX4‐Binder 5 complex exhibited conformations consistent with thermodynamically stable states of native proteins, showing no significant steric clashes, and glycine conformations displayed no abnormal constraints (Figure S6). The Free Energy Landscape (FEL), projected onto RMSD (PC1) and Rg (PC2), showed that the system attained minimum energy at an RMSD of approximately 0.17 and an Rg of approximately 1.60 (Figure 2H). The top candidates exhibited high predicted binding affinities and complementary interfaces with GPX4, establishing a solid foundation for the subsequent construction of bdC.

To experimentally validate the binding affinity of Binders 1–5 to GPX4, we synthesized N‐terminal biotinylated versions of these binders and characterized them using mass spectrometry and high‐performance liquid chromatography (Figures S8–S12). Cellular Thermal Shift Assay results demonstrated that while the GPX4 signal in the PBS control group nearly vanished at 62°C, high‐intensity bands were retained in the Binder 3, Binder 4, and Binder 5 groups. These findings indicate that all three compounds successfully bind to GPX4 and enhance its thermal stability (Figure 2I). Furthermore, enzyme‐linked immunosorbent assay analysis revealed that Binder 5 exhibited the optimal binding affinity for GPX4 (Figure 2J,K and Figure S13). To quantitatively assess the binding strength of our top candidates, we performed surface plasmon resonance (SPR) assays on Binder4 and Binder 5 (the two highest‐performing binders identified by CETSA and ELISA). SPR analysis revealed concentration‐dependent binding for both peptides. Binder 5 exhibited a relatively fast association rate (ka = 1.70 × 10^5^ M^−^^1^·s^−^^1^) and a measurable dissociation rate (kd = 2.10 × 10^−^^1^ s^−^^1^), yielding a kinetic equilibrium dissociation constant KD = 1.23 µM (steady‐state KD = 4.67 µM). This indicates low‐micromolar direct binding of binder5 to GPX4. Binder 4 showed a slower association (ka = 3.33 × 10^3^ M^−^^1^·s^−^^1^) but a more stable complex with lower dissociation (kd = 1.34 × 10^−^^2^ s^−^^1^), resulting in a kinetic KD = 4.04 µM (steady‐state KD = 7.20 µM). Notably, binder5 demonstrated a higher overall binding affinity to GPX4 compared to binder4, consistent with its superior functional activity observed in our cellular assays (Figure 2L–O). These biophysical data corroborate the ELISA and CETSA results and confirm that the de novo‐designed binders engage GPX4 with low‐micromolar affinity.

### 2.2 Construction of Binder‐Degron Chimeras

**FIGURE 3:**
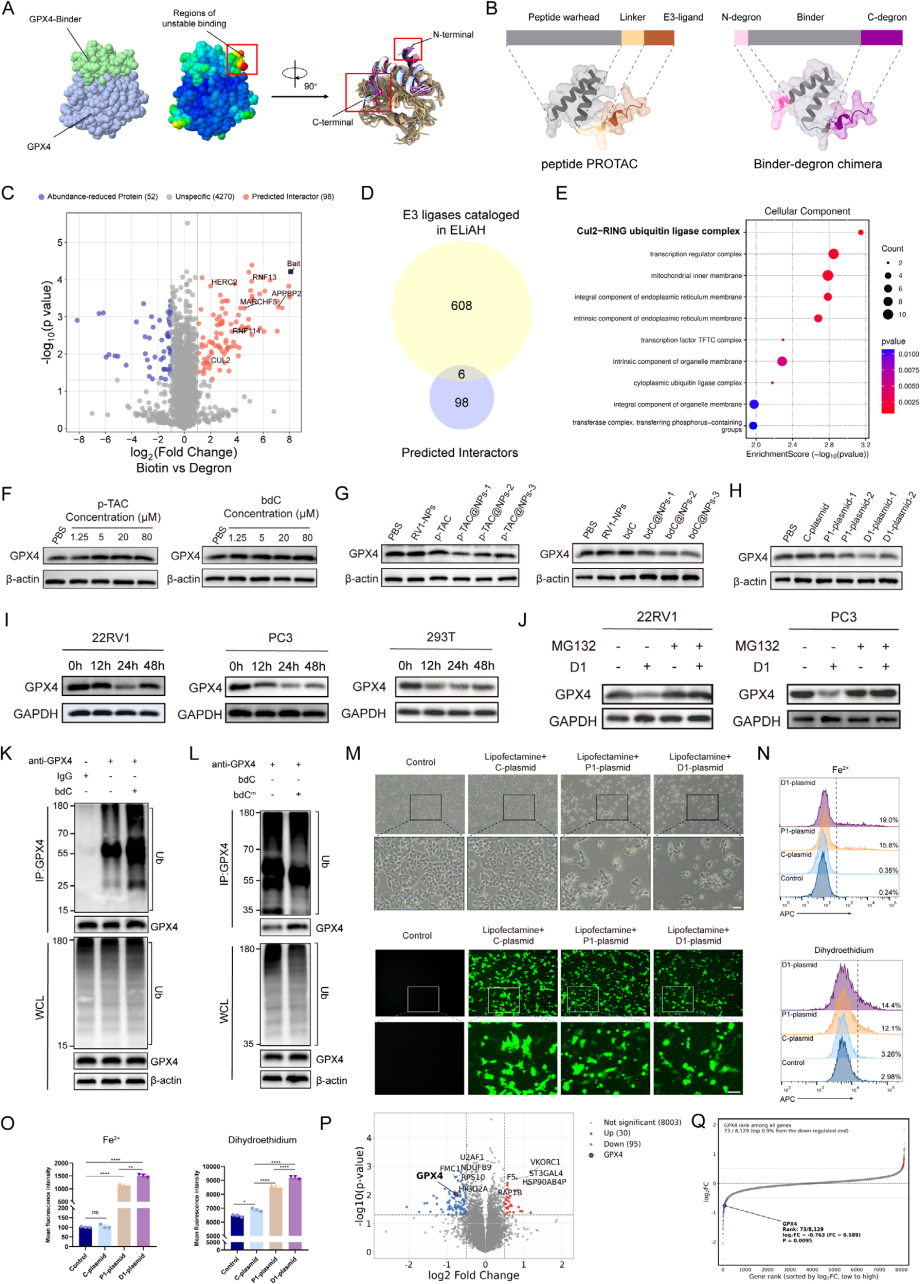
Binder‐degron chimeras degrade GPX4 via the proteasome and induce ferroptosis in prostate cancer cells. (A) Structural dynamics analysis showing the instability of the Binder5 C‐terminus. (B) Schematic of the construction of peptide PROTACs (p‐TAC) and binder‐degron chimeras (bdC) at the C‐terminus. (C) AP‐MS analysis identifying 98 predicted interactors recruited by the C‐degron. (D) Venn diagram showing the intersection between the predicted interactors and the E3 ligases cataloged in the ELiAH database, identifying 6 distinct E3 ligases. (E) Gene Ontology enrichment analysis of cellular components for the identified potential interacting proteins. The dot size represents the gene count, and the color indicates the p‐value. The results highlight the enrichment of the Cul2‐RING ubiquitin ligase complex. (F) Western blot analysis assessing GPX4 protein levels following treatment with indicated concentrations (1.25, 5, 20, and 80 µM) of p‐TAC and bdC. No dose‐dependent degradation was observed. β‐actin was used as an internal control. (G) Western blot analysis comparing the GPX4 degradation efficiency of free peptides (p‐TAC and bdC) versus their nanoparticle‐encapsulated formulations (p‐TAC@NPs and bdC@NPs). Evaluation of efficacy using different peptide‐to‐NP mass ratios: 1:10 (‐1), 1:20 (‐2), and 1:40 (‐3). Treatments were normalized to 1 µg/mL p‐TACs/bdCs (e.g., group ‐1 corresponds to 10 µg/mL peptide + 100 µg/mL MNPs). PBS and empty 22RV1‐derived nanoparticles (RV1‐NPs) served as controls. (H) Analysis of GPX4 protein levels in cells transfected with control plasmid (C‐plasmid), p‐TAC‐encoding plasmid (P1‐plasmid), or bdC‐encoding plasmid (D1‐plasmid). Transfection was performed on 5 × 10^5^ cells using 2.5 µg of plasmid and 4 µl of Lipofectamine. (I) Time‐dependent degradation of GPX4 in 22RV1, PC3, and HEK293T cells (0–48 h post‐transfection). (J) MG132 treatment rescues bdC‐induced GPX4 degradation, indicating a proteasome‐dependent pathway. (K‐L) 22RV1 cells were treated with D1 or C1‐plasmid (24 h), with MG132 (5 µM, 6 h) added before harvest to block proteasomal degradation. Cell lysates were immunoprecipitated with an anti‐GPX4 antibody (normal IgG as negative control), followed by immunoblotting with an anti‐ubiquitin (Ub) antibody. Immunoprecipitated GPX4 is shown as a loading control. Ubiquitination assays were performed to assess the effect of bdC expression on GPX4 ubiquitination (K) and to compare the effects of bdC and its degron‐mutant form (bdC^m^) (L). (M) Representative bright‐field and fluorescence images of transfected 22RV1 cells (Scale bar 20 µm). (N) Flow cytometric quantification of intracellular Fe^2+^and ROS levels (indicators of ferroptosis) in 22RV1 and PC3 cells expressing p‐TAC (P1‐plasmid) or bdC (D1‐plasmid). (O) Quantitative analysis of intracellular Fe^2^^+^ and ROS levels (Mean ± SD from three biologically independent experiments. One‐way ANOVA with Tukey's post hoc test). (P) Quantitative proteomic analysis of 22RV1 cells transfected with D1‑plasmid encoding bdC (n = 8136 proteins quantified; significance threshold: log_2_FoldChange < −0.5, p < 0.05). (Q) Gene‑rank plot illustrating the rank, log_2_ fold‑change, and P‑value of GPX4 among all detected genes in the proteomic dataset. ^*^ *p* < 0.05, ^**^ *p* < 0.01, ^***^ *p* < 0.001, ^****^ *p* < 0.0001.

Analysis of the binding mode between Binder 5 and GPX4 revealed that the C‐terminus of Binder 5 exhibited greater flexibility and fewer hydrogen bond interactions (Figure 3A). Consequently, we constructed a conventional peptide‐PROTAC (p‐TAC) by conjugating GPX4‐Binder 5 to the peptide ligand of the E3 ligase VHL (ALAPYIP) via a GGSGG linker. Additionally, we designed bdC by attaching a combinatorial degron to the C‐terminus of the binder (Figure 3B). The C‐terminal degron sequence was engineered as RFLGGREE, a sequence designed to simultaneously satisfy the RXXG, ‐RXX, and ‐EE degron motifs without mutual interference. Affinity Purification‐Mass Spectrometry (AP‐MS) analysis indicated that this degron sequence potentially interacted with 98 proteins. Cross‐referencing the proteins enriched by AP‐MS with the 614 E3 ligases cataloged in the ELiAH database identified six overlapping E3 ligases: APPBP2, MARCHF5, HERC2, RNF114, RNF13, and CUL2 (Figure 3C,D). Gene Ontology (GO) enrichment analysis further revealed a significant enrichment of the Cul2‐RING ubiquitin ligase complex (Figure 3E). However, we observed that direct cellular treatment with p‐TAC or bdC failed to induce GPX4 degradation at concentrations of 1.25, 5, 20, or 80 µM (Figure 3F).

### 2.3 Intracellular Expression of bdC Induces GPX4 Degradation

We observed that FITC‐labeled p‐TAC and bdC, being long‐chain peptides with molecular weights of approximately 6000 Da, exhibited poor cell membrane permeability. Fluorescence microscopy revealed that these peptides formed aggregates within the culture medium, indicating that a drug delivery system was required to facilitate intracellular internalization. Co‐extrusion of FITC‐modified p‐TAC and bdC with 22RV1 cell membrane‐derived nanoparticles resulted in a more diffuse intracellular distribution of the fluorescently labeled peptides (Figure S14). We prepared formulations with mass ratios of 1:10, 1:20, and 1:40 (designated as p‐TAC/bdC@NPs‐1, ‐2, and ‐3). Specifically, the ‐1‐group contained 10 µg/mL p‐TAC/bdC and 100 µg/mL MNPs, with the other groups scaling the peptide concentration accordingly while maintaining constant p‐TAC/bdC levels (Figure 3G).

Given the complexity of chemically synthesizing polypeptide chains exceeding 50 amino acids in vitro, and inspired by mRNA vaccine technology and the bioPROTAC concept, we engineered plasmids encoding p‐TAC and bdC. These were designated as P1‐plasmid and D1‐plasmid, respectively, and designed to achieve transient expression in prostate cancer cells. Transfection of 5 × 10^5^ cells with 2.5 µg of D1‐plasmid and 4 µL of Lipofectamine (D1‐plasmid‐1) yielded superior GPX4 degradation compared to the same conditions using the P1‐plasmid (Figure 3H). Transfection efficiency was monitored via GFP expression, reaching a peak 24 h post‐transfection with the D1‐plasmid. Assuming simultaneous expression of the chimeric peptides, this time point was designated as 0 h for subsequent analyses. We observed that GPX4 levels began to decline at 12 h in 22RV1, PC3, and 293T cells, with significant degradation evident by 24 h (Figure 3I).

To investigate the molecular mechanism underlying bdC‐mediated GPX4 degradation, we evaluated the involvement of the ubiquitin‐proteasome system (UPS), the major intracellular pathway for selective protein degradation. Subsequently, at 24 h post‐transfection with the D1 plasmid, the cells were incubated with 5 µM MG132 (a potent proteasome inhibitor) for an additional 6 h. While MG132 alone had no discernible effect on basal GPX4 levels, it effectively reversed bdC‐induced GPX4 depletion in both 22RV1 and PC3 cell lines, thereby indicating that bdC mediates GPX4 elimination via a proteasome‐dependent mechanism (Figure 3J). Additionally, we performed ubiquitination assays in 22RV1 cells. GPX4 was immunoprecipitated with an anti‐GPX4 antibody, with normal IgG serving as a negative control, and its ubiquitination status was examined by immunoblotting with an anti‐ubiquitin antibody. Smears (high molecular weight) characteristic of polyubiquitinated GPX4 were detected in GPX4 immunoprecipitates, with the most abundant species between 55 and 70 kDa. BdC expression markedly enhanced this smear, while no obvious changes were observed in the amount of immunoprecipitated GPX4 or in total ubiquitin levels in whole‐cell lysates. These results indicate that bdC promotes the polyubiquitination of GPX4 (Figure 3K). Unlike bdC, the degron‐mutant form bdC (GPX4 binder‐**RFGLGEER**, bdC^m^) failed to increase GPX4 polyubiquitination. This indicates that an intact degron is required for bdC‐mediated ubiquitination of GPX4 (Figure 3L).

Under bright‐field microscopy, the cells exhibited a shrunken and rounded morphology, fluorescence microscopy further revealed that cells transfected with P1 and D1 plasmids displayed green fluorescence, with the majority exhibiting this characteristic shrunken and rounded phenotype (Figure 3M). Compared to the untreated control and cells transfected with the control plasmid (C1‐plasmid), intracellular levels of Fe^2+^ and reactive oxygen species (ROS) were significantly elevated in cells transfected with P1 and D1 plasmids (Figure 3N,O).

To explore the global protein alterations triggered by bdC expression, we performed quantitative proteomic analysis on 22RV1 cells transfected with the D1 plasmid encoding bdC. Among the total quantified proteins, GPX4 exhibited significant downregulation upon bdC expression, with a log_2_foldChange of −0.763 (fold change = 0.589, p = 0.0095). Using a threshold of log_2_FoldChange < −0.5 as the cutoff, a total of 95 proteins were identified as significantly down‑regulated (Figure 3P). Further gene‑rank analysis revealed that GPX4 ranked 73 out of 8129 quantified proteins, placing it within the top 0.9% of down‑regulated proteins across the whole proteome dataset (Figure 3Q). Collectively, these proteomic data demonstrated that bdC overexpression markedly reduced GPX4 protein abundance in 22RV1 cells. Of note, apart from GPX4, numerous additional proteins were down‑regulated, which raises the possibility of off‑target effects induced by bdC.

### 2.4 Construction of Prostate Cancer‐Targeted Nanocarriers for bdC Delivery

**FIGURE 4:**
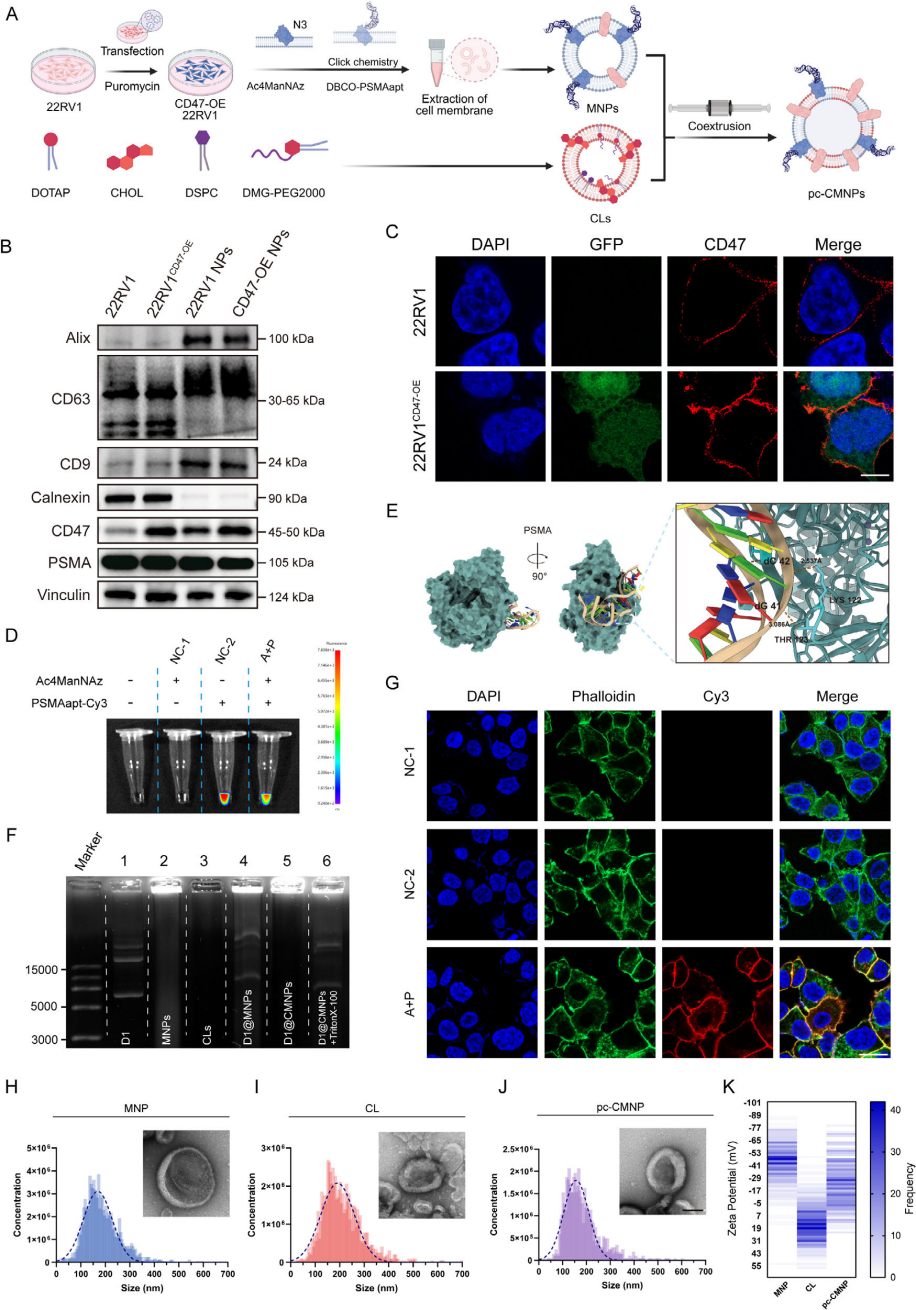
Design, Construction and Validation of PSMA‐targeted, CD47‐expressing, cationic liposome (CL)‐incorporated cell membrane‐derived nanoparticles (pc‐CMNP). (A) Component elements and construction process of pc‐CMNP. (B) Western blot analysis of membrane‐derived nanoparticles (NPs) from 22RV1 and CD47‐overexpressing (CD47‐OE) 22RV1 cells. Successful isolation of NPs was confirmed by the presence of exosomal markers (Alix, CD63, and CD9) and the absence of the endoplasmic reticulum marker calnexin. CD47 overexpression was verified in both source cells and isolated NPs. CD47 overexpression did not compromise the expression of PSMA in 22RV1 cells and 22RV1‐derived nanoparticles. (C) Confocal microscopy images of CD47 overexpressed on the cell membrane of 22RV1 cells (Scale bar 5 µm). (D) Cy3 fluorescence intensity in the culture supernatant of negative control groups (NC‐1, NC‐2) and the group cultured with Ac_4_ManNAz for 72 h, followed by addition of DBCO‐PSMAapt‐Cy3 (A+P) after 1 h of click reaction. (E) Schematic diagram of PSMA aptamer docking with PSMA. (F) Gel retardation assay for evaluating loading efficiency of various nanosystems on D1 plasmids (D1: D1 plasmid; MNPs: 22RV1 membrane‐derived nanoparticles; CL: cationic liposomes; D1@MNPs: co‐extrusion of D1 and MNPs with a 200 nm filter; D1@CMNPs: co‐extrusion of D1 and CMNPs with a 200 nm filter). (G) Confocal microscopy showed that only the A+P group exhibited Cy3 colocalization with the cell membrane (Scale bar 10 µm). Transmission Electron Microscopy (TEM) and Nanoparticle Tracking Analysis (NTA) of MNPs (H), CL (I), and pc‐CMNP (J) (Scale bar 100 nm). (K) Zeta Potentials of MNPs, CL, and pc‐CMNP.

To construct the delivery system, we transduced 22RV1 prostate cancer cells with a lentivirus capable of CD47 overexpression and selected for stable expression using puromycin, thereby establishing the CD47‐OE 22RV1 cell line. Following metabolic labeling with 25 µM Ac4ManNAz for 72 h, the cells were incubated with a 5'‐DBCO‐modified PSMA‐targeting aptamer for 1 h to facilitate a click chemistry reaction. Subsequently, cell membranes were isolated to yield CD47‐overexpressing, PSMA aptamer‐functionalized 22RV1 cell membrane‐derived nanoparticles (MNPs). To enhance the plasmid loading efficiency of the drug delivery system, we prepared cationic liposomes (CLs) using DOTAP, CHOL, DSPC, and DMG‐PEG2000, and co‐extruded these CLs with the MNPs to form pc‐CMNPs (Figure 4A).

For validation of the pc‐CMNPs, western blotting analysis indicated that protein levels of the exosome markers Alix, CD63, and CD9 were enriched in the extracted 22RV1 NPs and CD47‐OE 22RV1 NPs, while Calnexin levels were diminished. Additionally, elevated CD47 protein expression was confirmed in the lentivirus‐transduced cells, verifying the isolation of CD47‐overexpressing cell membrane‐derived nanoparticles, while PSMA expression in both 22RV1 cells and the derived nanoparticles remained unaffected (Figure 4B). Transduction efficiency was assessed via GFP fluorescence (Figure S15), Confocal fluorescence microscopy further revealed that the overexpressed CD47 was localized to the cell membrane (Figure 4C).

We performed a 3D reconstruction of the PSMA aptamer using 3dDNA and AlphaFold3 (Figure S16) and conducted molecular docking analysis to visualize the three‐dimensional interaction interface between the aptamer and the PSMA protein (Figure 4E). A PSMA‐targeting aptamer modified with DBCO‐TEG at the 5' end and Cy3 at the 3' end was synthesized (Figure S17). Following a 1 h click chemistry reaction between 10 µM DBCO‐PSMAapt‐Cy3 and Ac4ManNAz‐treated cells in 6‐well plates, we observed that the Cy3 fluorescence intensity in the supernatant of the non‐Ac4ManNAz‐labeled group was higher than that of the Ac4ManNAz‐labeled group. This depletion indicated that the PSMA aptamers significantly bound to the Ac4ManNAz‐labeled cells (Figure 4D). Confocal fluorescence microscopy confirmed that Cy3‐labeled PSMA aptamers were conjugated to the cell membrane of Ac4ManNAz‐labeled 22RV1 cells via click chemistry (Figure 4G and Figure S18). To confirm that azide groups remained intact throughout the nanocarrier preparation process, Ac4ManNAz‐labeled cell membranes were subjected to repeated freeze–thaw cycles and membrane extrusion to generate ∼200 nm nanoparticles. The resulting azide‐presenting nanocarriers were then incubated with DBCO‐PSMAapt‐Cy3 and counterstained with the lipophilic dye DiO. Confocal fluorescence microscopy revealed clear colocalization of Cy3 (red) and DiO (green) signals on the nanocarrier surface, indicating that the azide moieties survived freeze–thaw lysis and extrusion and remained accessible for efficient copper‐free click‐chemistry‐mediated PSMA aptamer conjugation (Figure S19A). Furthermore, to assess the colloidal stability of pc‐CMNPs under physiological conditions, we incubated the nanoparticles in cell culture medium supplemented with 10% FBS and monitored their hydrodynamic diameter and polydispersity index (PDI) over 72 h. Dynamic light scattering analysis showed that the mean particle size remained stable at approximately 180–190 nm, with no significant aggregation observed across all time points. Concurrently, the PDI values stayed consistently low throughout the 72 h period (Figure S19B,C).

Gel electrophoresis assays demonstrated that MNPs incorporating cationic liposomes (CMNPs) exhibited superior plasmid DNA loading and adsorption capabilities compared to MNPs alone (Figure 4F). The N/P ratio was calculated as the molar ratio of protonatable nitrogen atoms in DOTAP (MW = 698.5 g/mol, one nitrogen per molecule) to phosphate groups in the D1‐plasmid (330 g/mol per phosphate). We systematically screened three formulations with fixed plasmid input (2 µg) and increasing DOTAP content (5, 10, and 20 µg), yielding N/P ratios of 1.2, 2.4, and 4.7, respectively. Agarose gel electrophoresis retardation assays showed that free D1‐plasmid migrated readily into the gel, whereas D1@CMNPs‐1 (N/p = 1.2) and D1@CMNPs‐2 (N/p = 2.4) exhibited only partial retention in the loading well, indicating incomplete encapsulation. At N/p = 4.7 (D1@CMNPs‐3), the plasmid band was fully retained, with encapsulation efficiency exceeding 90% as quantified by densitometry. Furthermore, disruption of D1@CMNPs‐3 with Triton X‐100 restored the plasmid band, confirming that the absence of migration reflected effective entrapment rather than DNA degradation (Figure S20). Transmission electron microscopy (TEM) imaging and Nanoparticle Tracking Analysis (NTA) revealed that the particle sizes of MNPs, CLs, and pc‐CMNPs were predominantly distributed around 150 nm (Figure 4H–J). The mean zeta potential of the MNPs was approximately −45 mV, whereas the CLs and pc‐CMNPs exhibited values of approximately +19 and −28 mV, respectively (Figure 4K).

### 2.5 pc‐CMNP Enhances Cellular Uptake and Tumor Accumulation In Vitro and In Vivo

**FIGURE 5:**
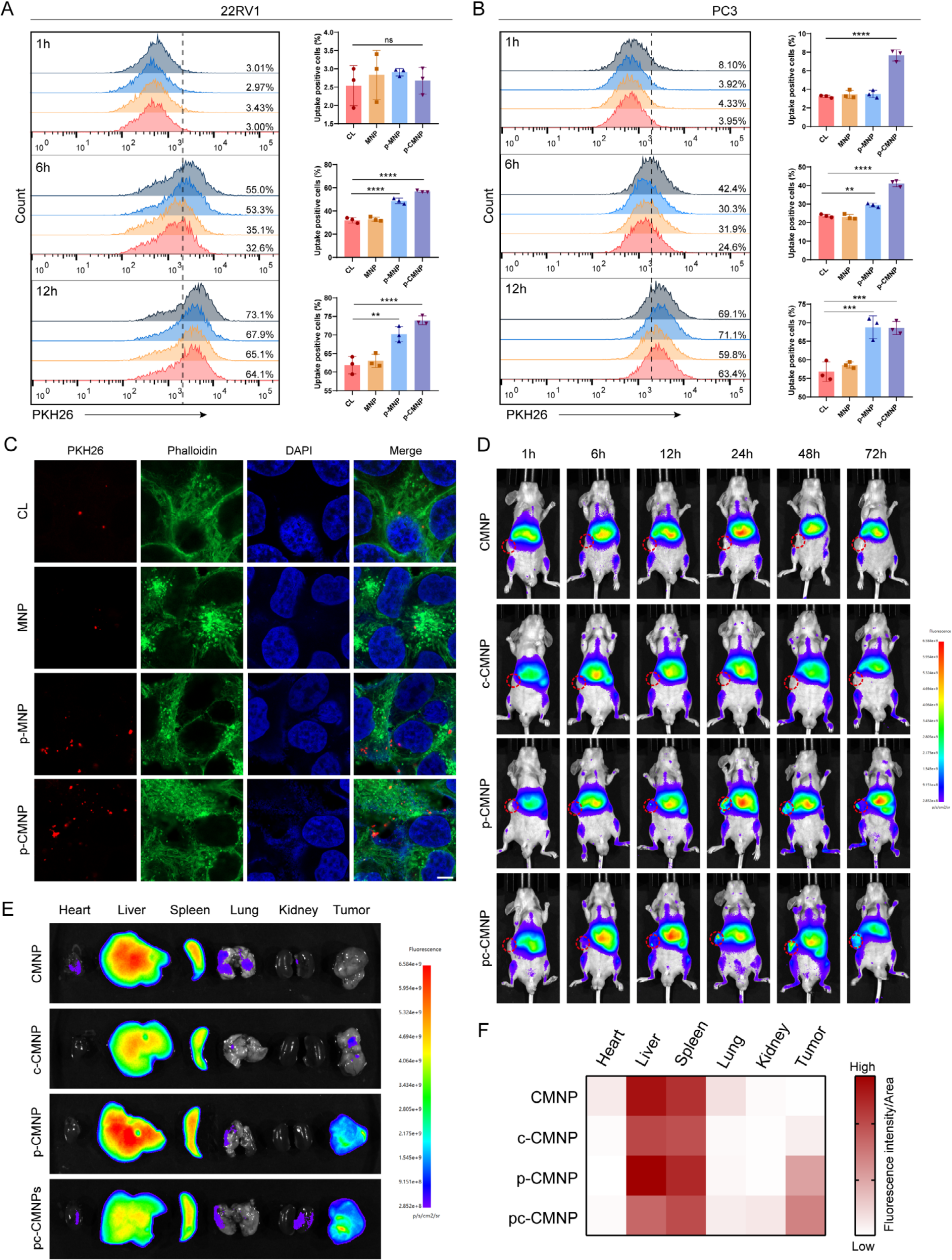
In vitro uptake and in vivo metabolism of different delivery system of D1. (A) Detection of intracellular uptake of PKH26‐stained cell membrane‐derived nanoparticles in 22RV1 cells and PC3 cells (B) by flow cytometry (Mean ± SD from three biologically independent experiments. One‐way ANOVA with Tukey's post‐hoc test). (C) Confocal microscopy of intracellular localization of PKH26‐stained nanoparticles (Scale bar 5 µm). (D) In vivo biodistribution of DiR‐stained CMNP, c‐CMNP, p‐CMNP, and pc‐CMNP in BALB/c nude mice over 72 h. (E) Ex vivo biodistribution of DiR‐stained nanoparticles in major organs and tumors of BALB/c nude mice. (F) Heatmap of DiR fluorescence intensity in major organs and tumors across CMNP, c‐CMNP, p‐CMNP, and pc‐CMNP groups. Abbreviations: CMNP: Cationic Liposome‐incorporated Cell Membrane‐derived Nanoparticles; c‐CMNP: Cationic Liposome‐incorporated CD47‐overexpressing Cell Membrane‐derived Nanoparticles; p‐CMNP: Click Chemistry‐modified PSMA Aptamer‐targeted Cell Membrane‐derived Nanoparticles. ^*^ *p* < 0.05, ^**^ *p* < 0.01, ^***^ *p* < 0.001, ^****^ *p* < 0.0001.

To evaluate the intracellular delivery efficiency of our system, we monitored the cellular uptake of CLs, c‐MNPs, pc‐MNPs, and the hybrid pc‐CMNPs (prepared by 1:1 co‐extrusion of CLs and pc‐MNPs) in 22RV1 and PC3 prostate cancer cells using flow cytometry. CLs, c‐MNPs, pc‐MNPs were administered at a standardized concentration of 10 µg/mL and incubated for 1, 6, and 12 h. In 22RV1 cells, we observed time‐dependent internalization, where the fluorescence intensity of pc‐MNPs and pc‐CMNPs significantly increased starting at 6 h compared to non‐targeted controls (Figure 5A), suggesting that PSMA aptamer functionalization effectively facilitates receptor‐mediated endocytosis. PC3 cells exhibited a robust uptake of pc‐MNPs and pc‐CMNPs at both 6 and 12 h (Figure 5B). Of note, PC3 cells are PSMA‑negative, and the prominent cellular uptake of pc‑CMNPs observed here might be ascribed to the intrinsic non‑specific endocytic activity of PC3 cells together with electrostatic interactions derived from the cationic liposome core, rather than PSMA‑dependent receptor‑mediated endocytosis. Confocal laser scanning microscopy visually confirmed that pc‐MNPs and pc‐CMNPs achieved superior cellular internalization, evidenced by a markedly higher density of PKH26‐labeled nanoparticles within the cytoplasm compared to non‐targeted groups (Figure 5C). Notably, the inclusion of cationic liposomes in the pc‐CMNP hybrid system did not compromise the active targeting efficiency of the PSMA aptamers.

We next assessed the biodistribution and tumor‐targeting capability of the nanoparticles in Balb/c nude mice bearing 22RV1 xenografts. Using the near‐infrared dye DiR for deep‐tissue imaging, we tracked the distribution of CMNPs, c‐CMNPs, p‐CMNPs, and pc‐CMNPs following tail vein injection. Non‐targeted CMNPs were predominantly sequestered by the reticuloendothelial system (RES), showing extensive accumulation in the liver and spleen with negligible tumor signal throughout the 0–72 h observation window. In contrast, c‐CMNPs exhibited reduced hepatic retention after 24 h, attributed to the “don't eat me” signaling of CD47. Crucially, formulations incorporating PSMA targeting (p‐CMNP and pc‐CMNP) began to accumulate in the tumor region as early as 6 h post‐injection, reaching peak intensity between 24 and 48 h (Figure 5D). Among all groups, the dual‐functionalized pc‐CMNPs demonstrated the most favorable biodistribution profile: significant tumor accumulation combined with reduced hepatic enrichment after 24 h compared to other formulations. Ex vivo imaging of major organs at 72 h further confirmed that pc‐CMNP treatment resulted in the highest tumor‐to‐background ratio and minimized off‐target accumulation in the liver and spleen (Figure 5E,F).

### 2.6 D1@Pc‐CMNP Induces Potent Cytotoxicity and Triggers Ferroptosis in Prostate Cancer Cells

To determine the optimal dose of D1@pc‐CMNP, 22RV1 cells were treated with escalating plasmid loads formulated at fixed or proportionally scaled nanoparticle amounts: control (a); 0.005 µg D1‐plasmid in 0.2 µg pc‐CMNP (b); 0.010 µg D1‐plasmid in 0.2 µg pc‐CMNP (c); 0.015 µg D1‐plasmid in 0.2 µg pc‐CMNP (d); 0.025 µg D1‐plasmid in 0.2 µg pc‐CMNP (e); and 0.050 µg D1‐plasmid in 0.4 µg pc‐CMNP (f). GFP fluorescence microscopy revealed that transfection efficiency increased in a dose‐dependent manner and plateaued at higher plasmid loads. Western blot analysis showed that GPX4 degradation correlated closely with transfection efficiency from formulations (b) to (e); however, further increasing the dose to formulation (f) did not produce appreciably stronger GPX4 degradation (Figure S21A–C). Concurrently, cell viability declined as the D1‐plasmid dose increased, particularly at the highest plasmid loading (f).

**FIGURE 6:**
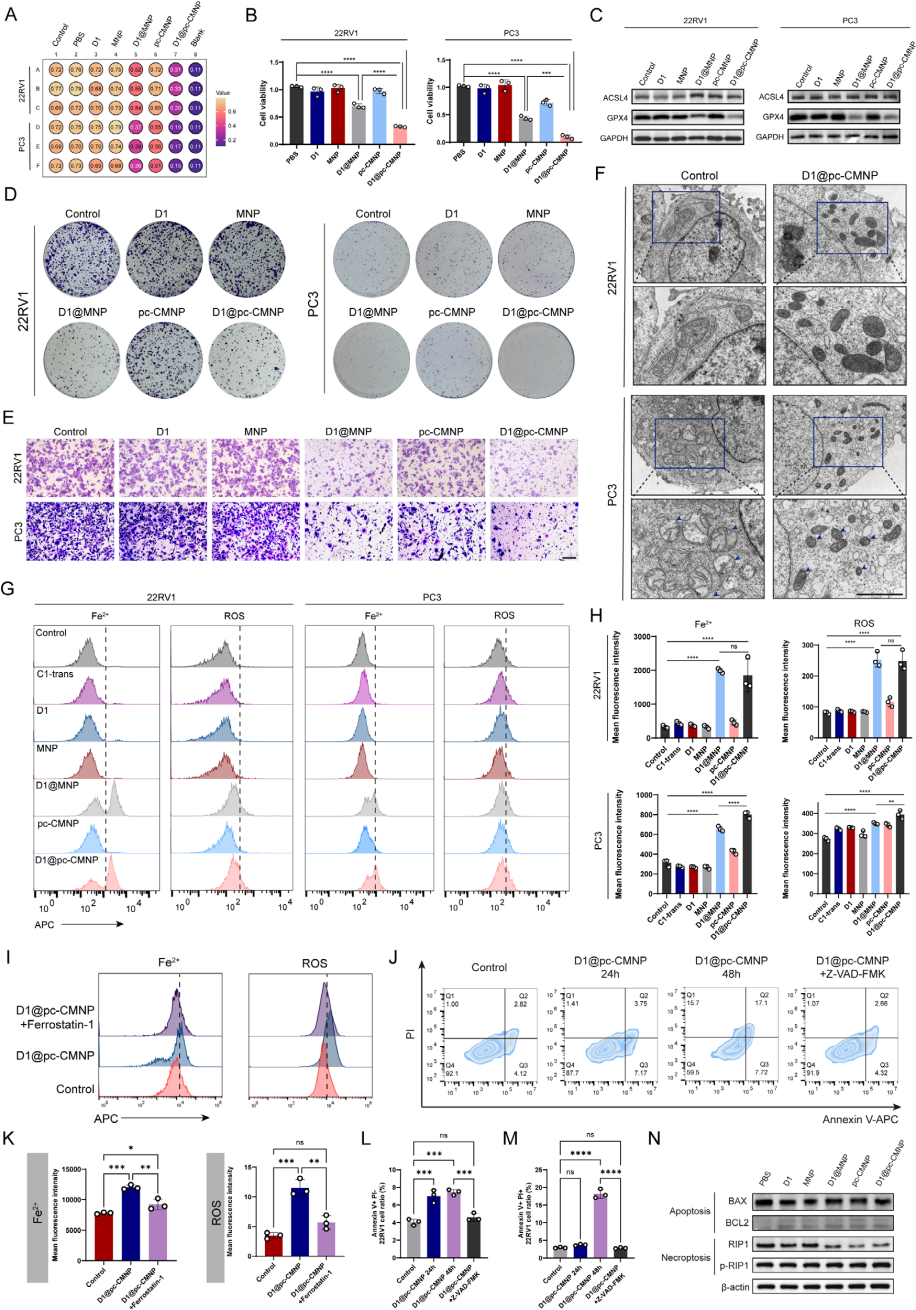
D1@MNP and D1@pc‐CMNP inhibit the proliferation and migration of 22RV1 and PC3 cells in vitro and induce ferroptosis in prostate cancer cells. (A) Cell viability of 22RV1 and PC3 cells treated with different formulations (Control, D1, MNP, D1@MNP, pc‐CMNP, D1@pc‐CMNP) was evaluated by a CCK‐8 assay. (B) Quantitative analysis of the CCK‐8 assay. (C) Western blot analysis of GPX4 and ACSL4 in 22RV1 and PC3 cells. (D) Colony formation capacity of 22RV1 and PC3 cells treated with D1‐loaded nanodelivery systems. (E) Migration capacity of 22RV1 and PC3 cells treated with D1‐loaded nanodelivery systems (Transwell assay; Scale bar 20 µm). (F) TEM images showing the ultrastructural changes in 22RV1 and PC3 cells. In D1@pc‐CMNP‐treated cells, mitochondrial shrinkage (marked by blue arrows) is observed, which is a typical morphological feature of ferroptosis (Scale bar, 1 µm). (G) Flow cytometry histograms of Fe^2^^+^ and ROS levels in 22RV1 and PC3 cells. (H) Flow cytometric analysis of Fe^2^^+^ and ROS levels in 22RV1 and PC3 cells. (I) Representative flow cytometry histograms of intracellular Fe^2^^+^ and ROS in 22RV1 cells treated with D1@pc‐CMNP in the presence or absence of ferrostatin‐1 (10 µM). (J) Flow cytometry of cell apoptosis. (Annexin V‐APC/PI staining; Q1, PI^+^ Annexin V^−^; Q2, PI^+^ Annexin V^+^, late‑apoptosis; Q3, PI^−^ Annexin V^+^, early‑apoptosis; Q4, PI^−^ Annexin V^−^).D1@pc‑CMNP induced a modest but statistically significant increase in early apoptosis, which was partially rescued by Z‑VAD‑FMK (20 µM). (K) Quantification of mean fluorescence intensity (MFI) for Fe^2^^+^ and ROS from flow cytometry. (L, M) Quantification of early apoptotic (Annexin V^+^ PI^−^, L) and late apoptotic (Annexin V^+^ PI^+^, M) populations. (N) Western blot analysis of apoptosis markers (BAX and BCL2) and necroptosis markers (RIP1 and p‐RIP1). Data are presented as mean ± SD from three biologically independent experiments (one‐way ANOVA, ^*^ *p* < 0.05, ^**^ *p* < 0.01, ^***^ *p* < 0.001, ^****^ *p* < 0.0001).

We performed CCK‐8 cell viability assays to evaluate the cytotoxicity of PBS, D1‐plasmid, MNP, D1@MNP, pc‐CMNP, and D1@pc‐CMNP treatments against tumor cells. The D1‐plasmid group was treated with 0.025 µg per 5 × 10^3^ cells, while the MNP and pc‐CMNP groups received 0.2 µg per 5 × 10^3^ cells. For the D1@MNP and D1@pc‐CMNP groups, prepared by co‐extruding plasmids (10 µg/mL) and nanoparticles (80 µg/mL), cells were treated with 0.2 µg of nanoparticles (containing an equivalent plasmid load) per 5 × 10^3^ cells (Figure 6A). In 22RV1 cells, D1@MNP reduced cell viability by approximately 35%, whereas D1@pc‐CMNP achieved a reduction of nearly 70%. In PC3 cells, D1@MNP decreased cell viability by roughly 55%, while D1@pc‐CMNP reduced it by approximately 90% (Figure 6B). Consistent with these findings, the D1@MNP and D1@pc‐CMNP groups showed significantly reduced colony formation (Figure 6D) and inhibited cell migration capabilities in both 22RV1 and PC3 cells (Figure 6E).

We next verified that D1@MNP and D1@pc‐CMNP induced ferroptosis in 22RV1 and PC3 cells. Western blot analysis showed that both treatments triggered the degradation of GPX4 in both cell lines. In addition to GPX4, we examined ACSL4, a core enzyme regulating ferroptotic lipid metabolism, finding it upregulated by D1@MNP and D1@pc‐CMNP in 22RV1 and PC3 cells. We posit that following GPX4 degradation, initial lipid peroxidation products begin to accumulate. ACSL4 incorporates free polyunsaturated fatty acids into membrane phospholipids via the Lands cycle, representing a lipid metabolic shift in response to the oxidative stress induced by GPX4 depletion (Figure 6C and Figure S22). Moreover, MDA levels in the supernatants of 22RV1 and PC3 cells increased in the D1@MNP, pc‐CMNP, and D1@pc‐CMNP groups, indicating the accumulation of lipid peroxides. Transmission electron microscopy (TEM) revealed that mitochondria in 22RV1 cells treated with D1@pc‐CMNP exhibited indistinct cristae and increased membrane density, while PC3 cells displayed obvious mitochondrial shrinkage (Figure 6F). Compared to the control group transfected with a GFP‐expressing plasmid (C1‐trans), D1@MNP and D1@pc‐CMNP significantly elevated intracellular Fe^2+^and ROS levels in 22RV1 cells (Figure 6G,H). In PC3 cells, D1@MNP and D1@pc‐CMNP also increased Fe^2+^and ROS, although the elevation in ROS was relatively moderate. To further validate the ferroptosis mechanism, we performed a rescue experiment by ferrostatin‐1, a specific ferroptosis inhibitor. D1@pc‐CMNP markedly elevated both Fe^2^^+^ and ROS compared to the control, and these elevations were substantially abrogated by co‐treatment with ferrostatin‐1 (10 µM) (Figure 6I). The quantification of mean fluorescence intensity confirmed these trends (Figure 6K).

To explore whether D1@pc‐CMNP triggers other forms of cell death, we performed Annexin V‐APC/PI staining and Western blot analysis. Flow cytometry revealed that D1@pc‐CMNP treatment induced a modest increase in early apoptotic cells (Annexin V^+^ PI^−^) at both 24 and 48 h (Figure 6J,L). However, the proportion remained low (approximately 7%), suggesting that apoptosis is not the predominant mechanism. Late apoptotic cells (Annexin V^+^ PI^+^) showed no significant increase at 24 h, with a slight elevation observed only at 48 h (Figure 6J,M). Treatment with the pan‑caspase inhibitor Z‑VAD‑FMK (20 µM) partially rescued this apoptotic population, confirming a minor caspase‑dependent apoptotic component. However, the overall proportion remained low, indicating that apoptosis plays only a marginal role compared to ferroptosis. Western blot analysis showed no notable changes in apoptosis markers (BAX and BCL2) across treatment groups. Regarding necroptosis, although total RIP1 appeared reduced in some treatment groups, phosphorylated RIP1 (p‐RIP1) showed no increase, providing no evidence for RIP1 kinase activation or necroptotic signaling (Figure 6N). Collectively, these data indicate that neither apoptosis nor necroptosis constitutes a primary driver of D1@pc‐CMNP–mediated cell death, supporting ferroptosis as the dominant mechanism.

### 2.7 In Vivo Antitumor Efficacy and Biosafety Profile of D1‐Loaded Pc‐CMNP

**FIGURE 7:**
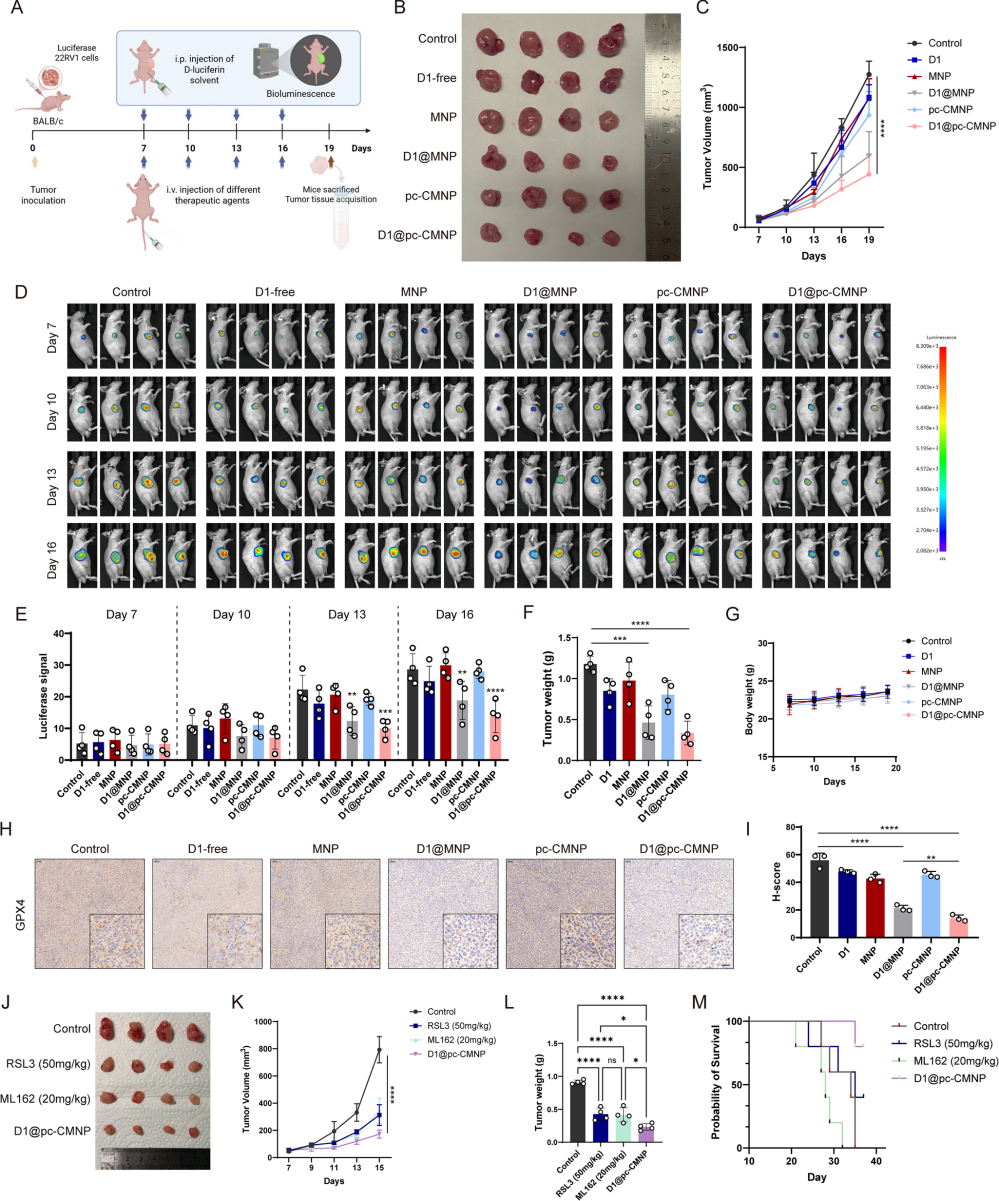
In vivo tumor growth inhibition and intratumoral GPX4 degradation by D1@pc‐CMNP. (A) Schematic of in vivo tumor treatment experimental procedure. (Administration was initiated on day 7 post‐tumor inoculation, with dosing every 3 days; potassium luciferin was intraperitoneally injected concurrently for in vivo imaging). (B) Photographs of excised tumors from different treatment groups on day 19. (C) Tumor growth curves following various treatments. Mice were treated with Control, D1, MNP, D1@MNP, PC‐CMNP, or D1@pc‐CMNP, and tumor volumes were measured every 2 days. Data are presented as mean ± SD and were analyzed using two‐way ANOVA (*p* < 0.0001 for D1@pc‐CMNP vs Control). (D) In vivo bioluminescence imaging of tumor‐bearing mice on days 7, 10, 13, and 16 post‐tumor inoculation. (E) Quantitative analysis of tumor bioluminescence intensity in each group (n = 4). (F) Tumor weights of different treatment groups (n = 4; mean ± SD; One‐way ANOVA). (G) Body weight changes of mice during treatment (n = 4). (H) Immunohistochemical staining of GPX4 in tumor tissues (Scale bar, 50 µm). (I) Quantitative analysis of GPX4 staining intensity in tumor tissues (IHC H‐score; n = 4; mean ± SD; one‐way ANOVA). (J) Nude mice bearing 22RV1 subcutaneous xenografts were treated with PBS (Control), RSL3 (50 mg/kg), ML162 (20 mg/kg), or D1@pc‐CMNP. Representative images of excised tumors (Day 15). (K) Tumor growth curves showing tumor volume over time; D1@pc‐CMNP inhibited tumor growth more potently than RSL3 (50 mg/kg, p = 0.0002) and ML162 (20 mg/kg, p < 0.0001). (L) Tumor weights; D1@pc‐CMNP significantly reduced tumor burden across all groups (n = 4). (M) Kaplan‐Meier survival analysis demonstrating prolonged overall survival in the D1@pc‐CMNP group relative to RSL3, ML162, and control groups (n = 5). Data are presented as mean ± SD. Statistical significance was determined by two‐way ANOVA with Tukey's post hoc test (K), one‐way ANOVA (L), and log‐rank test (M) ^*^ *p* < 0.05, ^**^ *p* < 0.01, ^***^ *p* < 0.001, ^****^ *p* < 0.0001.

To evaluate the in vivo therapeutic efficacy, we established a xenograft model by inoculating luciferase‐expressing 22RV1 cells into BALB/c nude mice. When subcutaneous tumors reached an average volume of approximately 100 mm^3^ (around day 7), we initiated treatment via tail vein injection every 3 days. Tumor growth dynamics were monitored continuously throughout the treatment period by in vivo bioluminescence imaging (BLI) following intraperitoneal injection of luciferin (Figure 7A). Compared to the control group, tumor volumes in the D1@MNP and D1@pc‐CMNP groups were reduced by an average of 54% and 64%, respectively (Figure 7B,C). BLI analysis further corroborated these findings: while bioluminescence signals in the control group increased exponentially, the D1@MNP and D1@pc‐CMNP treatments began to suppress tumor growth by day 10, achieving significant inhibition by day 16 (Figure 7D,E). At the experimental endpoint (day 19), tumors were excised and weighed. The average tumor weight in the D1@pc‐CMNP group was significantly lower than that in the Control and D1@MNP groups (Figure 7F), confirming the superior therapeutic efficacy of the complete delivery system. Importantly, no significant body weight loss was observed across any group during the treatment period (Figure 7G), indicating that D1@pc‐CMNP did not induce severe systemic toxicity. We next validated the induction of ferroptosis within the tumor tissue. Immunohistochemical (IHC) staining revealed GPX4 degradation in both the D1@MNP and D1@pc‐CMNP groups (Figure 7H), with H‐score statistical analysis confirming significantly lower GPX4 expression in the D1@pc‐CMNP group (Figure 7I).

To directly compare the therapeutic performance of our degrader‐based strategy with conventional GPX4 inhibitors, we established 22RV1 subcutaneous xenografts in nude mice and randomized them into four treatment groups: PBS control, RSL3 (50 mg/kg), ML162 (20 mg/kg), and D1@pc‐CMNP. Tumor growth monitoring over 15 days revealed that D1@pc‐CMNP potently suppressed tumor progression, achieving smaller tumor volumes than both RSL3 and ML162 (Figure 7J,K). Excised tumor weights in the D1@pc‐CMNP group were significantly lower than those in the control and inhibitor‐treated groups (Figure 7L). To assess long‐term therapeutic efficacy and the impact of drug treatment, we evaluated overall survival in nude mice (n = 5 per group). Notably, the RSL3 and ML162 groups showed limited survival benefit, likely reflecting the narrow therapeutic indices and systemic toxicities associated with conventional GPX4 inhibitors (Figure 7M). Collectively, these comparative data demonstrate that tumor‐targeted delivery of the bdC chimera via pc‐CMNP achieves superior antitumor efficacy and an improved safety profile relative to systemic administration of small‐molecule GPX4 inhibitors.

**FIGURE 8:**
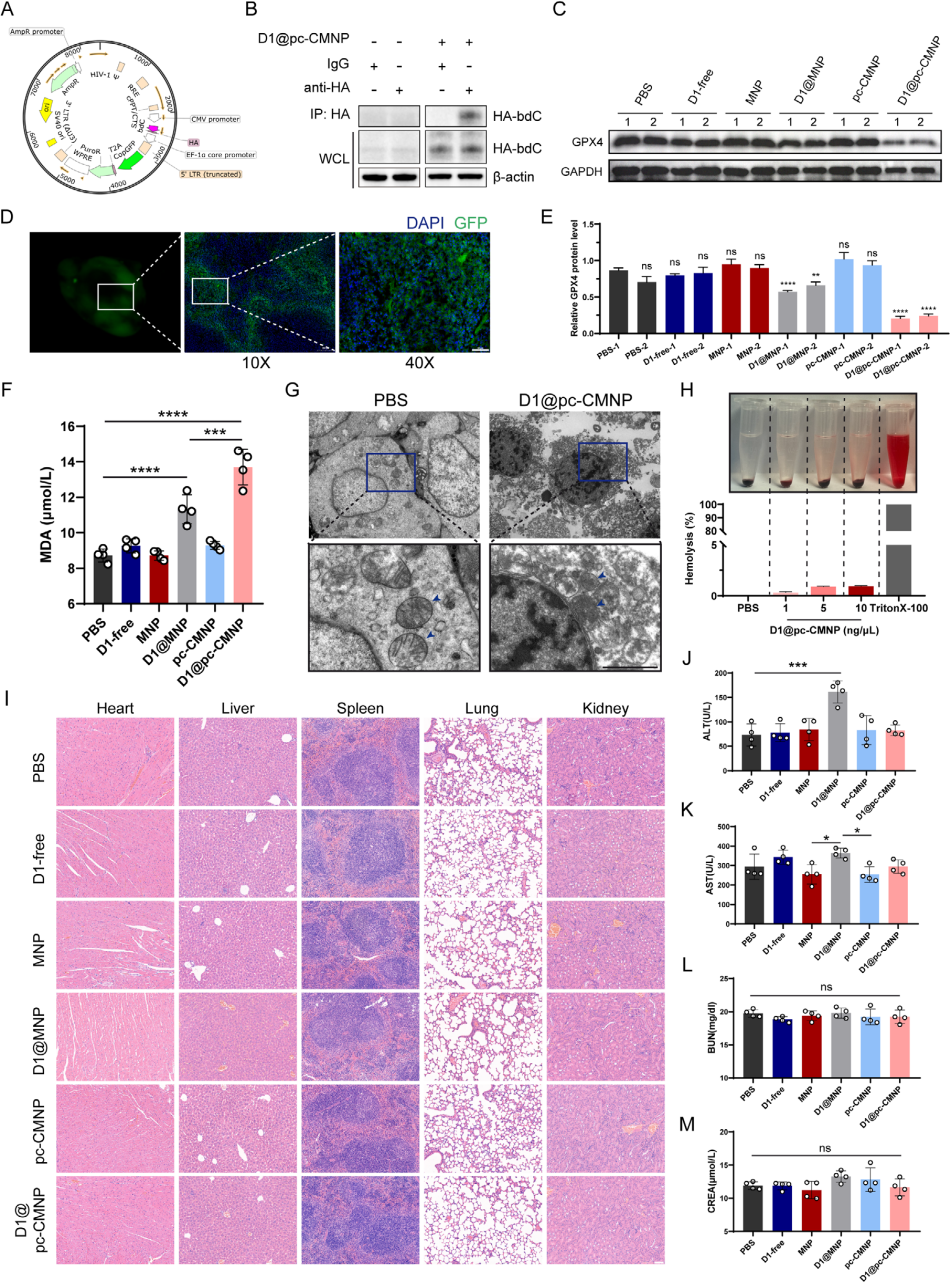
In vivo characterization of tumor ferroptosis markers and biosafety evaluation of D1@pc‐CMNP. (A) Schematic map of the plasmid for the detection of the expression of bdC. The construct contains two independent expression cassettes: a CMV promoter‐driven HA‐tagged binder‐degron chimera (HA‐bdC) and an EF1α promoter‐driven CopGFP‐T2A‐PuroR reporter cassette. (B) Validation of HA‐bdC expression by immunoprecipitation and immunoblotting. HA‐bdC was detected in both the immunoprecipitation (IP: HA) and whole‐cell lysate (WCL) fractions, confirming successful expression of the fusion protein. (C) Western blot analysis of GPX4 protein expression in tumor tissues harvested from mice treated with different formulations (PBS, D1‐free, MNP, D1@MNP, pc‐CMNP, D1@pc‐CMNP). (D) In vivo expression of the D1‐plasmid in tumor tissue. Nude mice bearing 22RV1 subcutaneous xenografts were administered D1@pc‐CMNP via tail vein injection. Tumors were harvested, snap‐frozen in liquid nitrogen, and sectioned for fluorescence microscopy. DAPI staining (blue) marks nuclei, and GFP fluorescence (green) confirms plasmid‐derived reporter expression within the tumor (Scale bar 50 µm). (E) Semi‐quantitative analysis of GPX4 protein levels (normalized to GAPDH, mean ± SD; one‐way analysis of variance). (F) Quantitative analysis of malondialdehyde (MDA) levels (nmol/L) in mouse orbital blood samples across treatment groups. (G) TEM images showing mitochondrial morphological changes in tumor tissues (blue arrows indicate shrunken mitochondria) from PBS‐ and D1@pc‐CMNP‐treated mice (Scale bar 1 µm). (H) Photographs of mouse orbital blood samples and quantitative analysis of hemolysis rate in different treatment groups (D1@pc‐CMNP at 1, 5, 10 ng/µL; PBS as negative control; Triton X‐100 used as positive control). (I) H&E staining of major organs from mice in each treatment group, assessing in vivo biosafety (Scale bar 50 µm). (J–M) Quantitative analysis of serum biochemical indicators for hepatic and renal function (ALT: alanine aminotransferase, AST: aspartate aminotransferase, BUN: blood urea nitrogen, CRE: creatinine) in mouse serum samples across treatment groups (mean ± SD). ^*^ *p* < 0.05, ^**^ *p* < 0.01, ^***^ *p* < 0.001, ^****^ *p* < 0.0001.

To validate the expression of bdC in vivo, we constructed a plasmid (pCDH‐CMV‐HA‐bdC‐EF1‐CopGFP‐T2A‐Puro), which harbors two independent transcriptional units: a CMV promoter‐driven HA‐tagged binder‐degron chimera (HA‐bdC) and an EF1α promoter‐driven CopGFP‐T2A‐PuroR cassette for tracking (Figure 8A). To confirm expression of the fusion protein, tumor lysates were subjected to immunoprecipitation using an anti‐HA antibody. Immunoblotting detected HA‐bdC in both the immunoprecipitated fraction and whole‐cell lysates, validating successful intracellular production of the bdC chimera (Figure 8B). To verify that the plasmid remains transcriptionally active in vivo, tumors were harvested, snap‐frozen, and sectioned for fluorescence microscopy. DAPI staining revealed intact tumor architecture, while robust GFP fluorescence was readily detected within the tumor tissue (Figure 8D). Western blot analysis of randomly selected tumors (two per group) demonstrated the most robust GPX4 degradation in the D1@pc‐CMNP group (Figure 8C,E). Furthermore, plasma analysis showed that malondialdehyde (MDA) levels were highest in mice treated with D1@pc‐CMNP (Figure 8F). At the ultrastructural level, TEM of tumor tissues revealed that cells in the D1@pc‐CMNP group exhibited characteristic ferroptotic features, including shrunken mitochondria and compromised cell membrane integrity with a ruptured morphology (Figure 8G).

Finally, we comprehensively assessed the biosafety profile of the plasmid‐loaded nanocomplexes. Hemolysis assays revealed acceptable hemocompatibility, with hemolysis rates remaining below 1% at a concentration of 1 ng/µL and approximately 2% at higher concentrations (5 and 10 ng/µL) (Figure 8H). Histological examination (H&E staining) of major organs, including the heart, liver, spleen, lung, and kidney, revealed no obvious morphological abnormalities (Figure 8I). Alanine aminotransferase (ALT) levels were elevated in the D1@MNP group (Figure 8J), whereas aspartate aminotransferase (AST) levels showed no significant anomalies across all groups (Figure 8K). Furthermore, blood urea nitrogen (BUN) and creatinine (CREA) levels remained within normal ranges, indicating no impairment of renal function (Figure 8L,M).

## 3 Discussion

In drug‐resistant cancers like castration‐resistant prostate cancer (CRPC), targeting GPX4 remains challenging [26]. Recent work has further demonstrated that a ferroptosis‐suppressive state drives resistance to conventional therapies in treatment‐refractory tumors such as muscle‐invasive bladder cancer, underscoring the translational necessity of reactivating ferroptosis to overcome drug resistance [27]. Traditional small‐molecule inhibitors often suffer from poor pharmacokinetics, lack of selectivity, or off‐target toxicity. In this study, we introduce a novel approach to target GPX4 without reliance on Sec46 or Cys66 residues [28, 29]. Instead, we employed an AI‐driven protein design method (Bindcraft) to generate specific binders that bind reversibly to GPX4. We successfully engineered a plasmid‐encoded bdC, which elicits potent GPX4 degradation via the proteasome system. By encapsulating the D1‐plasmid into a PSMA‐targeted, CD47‐overexpressing cell membrane‐lipid hybrid nanoparticle (pc‐CMNP), we achieved precision bdC expression in CRPC cells and significant antitumor efficacy in vivo, establishing a novel modality for ferroptosis‐based cancer therapy.

Our strategy begins with the de novo design of high‐affinity protein binders. In the field of drug discovery, multiple strategies for screening small‐molecule inhibitors against a given protein of interest (POI) have demonstrated superiority over conventional drug screening approaches [30]. Although library‐based virtual screening enables the identification of bioactive molecules, this method is often constrained by the limited availability of small‐molecule libraries [31]. In contrast, molecule generation technologies have advanced rapidly through deep learning frameworks such as graph neural networks (GNNs) and transformers, alongside breakthroughs in protein structure prediction like AlphaFold and RFdiffusion [6, 32, 33]. Consequently, these innovations have progressively facilitated the rational design and screening of bioactive molecules targeting POIs via a de novo generation paradigm. Here, we employed Bindcraft to generate binders targeting the surface cavities of GPX4. Our results demonstrated that the top candidate, Binder5, formed a stable complex with GPX4 through hydrogen bonds and hydrophobic interactions.

To convert this binder into a degrader, we bypassed the reliance on specific E3 ligase ligands by engineering a C‐terminal combinational degron. Current GPX4 degraders typically employ the covalent warhead RSL3 conjugated to ligands for established E3 ligases, such as CRBN. However, covalent modification of the GPX4 active site poses a risk of off‐target toxicity, necessitating the optimization of non‐covalent targeting strategies [34, 35]. Although the specific E3 ligase responsible for physiological GPX4 turnover and the endogenous degron sequences involved remain elusive [36], we hypothesized that appending a C‐terminal degron to our GPX4 binder could induce target instability [37, 38]. By forming a stable complex with GPX4, the binder effectively ‘tags’ the protein with a degron, enabling recognition by E3 ligases and triggering degradation via the proteasome system. Previous efforts utilizing degron‐based strategies have faced limitations: Qu et al. employed a peptide‐PROTAC with a C‐terminal RRRG motif to degrade α‐synuclein, yet this approach required high concentrations (∼50 µM) for efficacy [39]. Zhang et al. utilized single amino acids as potential degrons, though the specificity of such minimal motifs for E3 recruitment remains unclear [34]. In contrast, our proteomic analysis and affinity purification‐mass spectrometry (AP‐MS) data revealed that our engineered degron recruits six distinct E3 ligases and the CUL2‐RING ubiquitin ligase complex, driving robust GPX4 degradation. Importantly, despite carrying degradation signals, the bdC itself evades self‐degradation. We attribute this stability to the short length of the binder (50–60 amino acids), which is insufficient to serve as a substrate for ubiquitination [40].

Despite the evolution of peptide‐based TPD, conventional peptide‐PROTACs remain hindered by high molecular weight (>2 kDa), limited cell permeability, and complex chemical synthesis inherent in their chimeric design. To address these challenges, emerging strategies such as bioPROTACs and mRNA vaccines have demonstrated the potential of intracellularly expressing therapeutic proteins [41, 42]. More recently, Li et al. established a guided protein labeling and degradation system (GPlad) in Escherichia coli, leveraging de novo‐designed guide proteins and arginine kinase (McsB) to achieve precise degradation of diverse targets [43]. Inspired by these successes, we circumvented traditional limitations by encoding the bdC into a plasmid (D1‐plasmid), thereby enabling cancer cells to synthesize the degrader in situ via their endogenous expression machinery. This strategy of intracellular degrader expression presents distinct advantages and challenges. It enables the utilization of macromolecular binders, nanobodies, and even antibodies to engage proteins that remain difficult to translate clinically because of selectivity, pharmacokinetic, and systemic toxicity concerns. Additionally, this approach may also mitigate toxicity arising from the off‐target effects of small molecules. Furthermore, the cell‐autonomous production of active proteins effectively resolves the issues of poor membrane permeability, low bioavailability, and High immunogenicity that often plague macromolecular therapeutics [23, 44]. However, several limitations persist. The efficacy of the expressed degrader could be influenced by variable post‐translational modifications. Naked plasmids are vulnerable to nuclease degradation and inherently lack tumor specificity. Moreover, like any PROTAC system relying on ternary complex formation, this approach is susceptible to the “hook effect”, where excessive drug concentrations fail to induce degradation. Consequently, a current limitation of the cell‐autonomous strategy is the difficulty in precisely controlling the intracellular concentration of the bdC to avoid this effect [45].

The precise expression of bdCs within prostate cancer cells remains a critical challenge. Existing non‐viral vectors, such as cationic liposomes or polymers, often suffer from rapid clearance by the reticuloendothelial system. Our pc‐CMNP system addresses this by camouflaging the cationic liposome core with a CD47‐overexpressing cell membrane. For instance, Du et al. previously modified exosomes with the CD47‐derived targeting peptide (RLTRKRGLK) to significantly decrease macrophage‐mediated clearance [46]. This “don't eat me” signal significantly reduced hepatic accumulation compared to standard liposomes. Furthermore, to enhance selectivity, we integrated a high‐affinity PSMA‐targeting aptamer, originally developed by Deng et al. for prostate cancer diagnosis [47]. To anchor this high‐affinity aptamer onto the cell membrane‐derived vesicles, we employed bioorthogonal click chemistry between azide and DBCO groups [48]. To ensure the stable and dense presence of azide groups on the membrane surface, we adopted the metabolic engineering strategy described by Lamoot et al., utilizing Ac4ManNAz to facilitate the positioning of azide‐tagged mannose onto membrane glycoproteins [49]. Specifically, we modified the PSMA‐targeting aptamer with DBCO to react with the azide groups on the 22RV1 cell membranes. The incorporation of PSMA aptamers conferred active targeting capabilities superior to passive EPR effects alone. While direct evidence for plasmid escape from endosomes or lysosomes was not obtained, GFP expression driven by the plasmid was readily detected in the tumors of nude mice bearing 22RV1 subcutaneous xenografts after tail vein administration. The 22RV1 cell membrane coating on pc‐CMNPs promotes endosomal escape primarily through membrane fusion between the biomimetic membrane and the endosomal membrane. This hierarchical design, which integrates payload protection, macrophage evasion, and active tumor targeting, ensures that therapeutic concentrations of the bdCs are achieved specifically within CRPC cells.

Despite the encouraging therapeutic efficacy demonstrated in this study, several limitations should be acknowledged. First, although Binder5 exhibits high affinity and structural stability toward GPX4, its interaction interface is derived from in silico design and validation. Future work incorporating high‐resolution structural characterization, such as cryo‐electron microscopy or X‐ray crystallography of the GPX4‐binder complex, would further elucidate the precise binding mode and guide additional optimization of binder affinity and specificity. Despite validating the expression and functional effects of the plasmid‐encoded bdC, its post‐translational modification status was not explicitly confirmed through experimental assays. Furthermore, the transcriptional and translational efficiency of the bdC may vary across different cell types and tumor microenvironments [50]. In the context of targeted protein degradation, a more detailed dissection of E3 engagement and ubiquitination sites on GPX4 would deepen our mechanistic understanding and enable the rational selection or engineering of degrons with enhanced efficiency and reduced off‐target effects [51]. Furthermore, regarding the biomimetic membrane coating strategy, we acknowledge that lentiviral‐driven CD47 overexpression in 22RV1 cells may introduce complex alterations in the native membrane proteome beyond the scope of our PSMA validation, potentially affecting the biomimetic fidelity of the resulting pc‐CMNP. While extensive in vitro and in vivo analyses support ferroptosis as the dominant mode of cell death induced by D1‐mediated GPX4 degradation, we cannot fully exclude contributions from secondary stress responses, including oxidative damage‐induced apoptosis, metabolic collapse under certain conditions, and other cell death modalities [52]. Finally, we acknowledge that the subcutaneous tumor model does not fully recapitulate the metastatic characteristics of CRPC, which predominantly involves bone and visceral dissemination. Future studies will therefore focus on orthotopic prostate cancer models and established bone metastasis models to evaluate whether pc‐CMNPs can effectively target circulating tumor cells, bone marrow niches, and lymph node metastases following systemic administration.

In summary, our work explores a new paradigm in protein degradation by combining deep learning‐based binder design with the assembly of a unique binder‐degron chimera mode. By constructing a prostate cancer‐targeted nanomedicine system, we enable cancer cells to self‐express the bdC, thereby triggering targeted GPX4 degradation and ferroptosis. This approach overcomes the limitations of traditional small‐molecule inhibitors and establishes a robust platform for precision medicine, extending the therapeutic reach of TPD to previously “undruggable” intracellular targets and offering a promising solution for treating drug‐resistant cancers such as CRPC.

## 4 Materials and Methods

### 4.1 De Novo Design and Screening of GPX4 Binders

GPX4‐targeting mini‐binders were designed with the BindCraft protocol (GitHub: martinpacesa/BindCraft). The human GPX4 crystal structure (PDB ID: 6ELW) was imported into UCSF ChimeraX (https://www.cgl.ucsf.edu/chimerax/); crystallographic ligands and solvent molecules were removed, and the surface was visually inspected to select a solvent‐exposed, druggable epitope for binder engagement. To minimize molecular weight, the binder length was constrained to 10–70 residues. In total, 157 independent design trajectories were generated. Candidates were progressively filtered according to the BindCraft recommended criteria: AlphaFold2 pLDDT > 0.8, interface pTM (i_pTM) > 0.5, interface predicted aligned error (i_pAE) < 0.35, Rosetta interface shape complementarity > 0.55, and <3 unsatisfied hydrogen bonds. These thresholds yielded 21 high‐confidence designs. The 10 top‐ranking designs were subsequently submitted to H‐DOCK (http://hdock.phys.hust.edu.cn/) for protein‐protein docking and re‐scoring. Combining the BindCraft rankings with the H‐DOCK scores, we selected the five best‐scoring peptides for synthesis, denoted binder1–5.

### 4.2 Molecular Dynamics Simulation

A 100‐ns MD simulation of the complex was performed with GROMACS 2022. The protein‐binder complex was modeled using the AMBER99SB‐ILDN all‐atom force field. Periodic boundary conditions were applied by placing the complex in a cubic box, which was solvated with TIP3P water molecules to create a 1.2 nm hydration layer. Electrostatic interactions were treated with the particle‐mesh Ewald (PME) method and a Verlet cutoff scheme. The system underwent a two‐stage equilibration process: first, 100 000 steps in the canonical ensemble (NVT; constant particle number, volume, and temperature), followed by 100 ps in the isothermal‐isobaric ensemble (NPT; constant particle number, pressure, and temperature), both utilizing a 0.1 ps coupling constant. Van‐der‐Waals and Coulomb interactions were truncated at 1.0 nm. Finally, a 100‐ns production run was carried out at constant temperature (310 K) and pressure (1 bar) with GROMACS 2022.

### 4.3 Western Blot

Proteins were extracted from 22RV1 or PC3 cells using RIPA supplemented with protease inhibitors (PMSF) to prevent protein degradation. The protein concentration was determined using a BCA protein assay kit (P0009, Beyotime, China). For SDS‐PAGE, proteins were mixed with 5x loading buffer, boiled for 15 min at 95°C–100°C, and then separated on 10% SDS‐polyacrylamide gels at 80 V for 120 min. After electrophoresis, proteins were transferred onto PVDF membranes using a semi‐dry transfer apparatus (Bio‐Rad) at 220 mA for 65 min. The membranes were blocked with 5% non‐fat milk in TBST (Tris‐buffered saline with Tween‐20) for 1 h at room temperature to prevent non‐specific binding. The membranes were then incubated with primary antibodies against GPX4 (Abmart, China, dilution ratio 1:1000), ACSL4 (Abmart, China, dilution ratio 1:1000) overnight at 4°C. GAPDH (Cell Signaling Technology, dilution ratio 1:2000) and β‐actin (ABclonal, China, dilution ratio 1:10000) were used as internal controls to normalize the protein loading. After washing three times with TBST for 10 min each, the membranes were incubated with secondary antibodies conjugated with horseradish peroxidase (ABclonal, China, HRP‐conjugated goat anti‐rabbit IgG, dilution ratio 1:10000) for 1.5 h at room temperature. The protein bands were visualized using an ECL chemiluminescence detection kit (Yeasen Biotechnology, China) and imaged with a chemiluminescence imaging system. The band intensities were semi‐quantified using ImageJ 1.54i, and each experiment was repeated three times.

### 4.4 ELISA Assay for Binder Detection

The binding of GPX4 binder to GPX4 was determined using the Human Phospholipid Hydroperoxide Glutathione Peroxidase, Mitochondrial (GPX4) ELISA Kit (CSB‐EL009869HU, Cusabio, China). Biotinylated binders 1–5 (synthesized by QYAOBIO, China) were completely dissolved in PBS to final concentrations of 3.125, 6.25, 12.5, 25, 50, and 100 µg mL^−^^1^. An equal volume of each biotinylated binder was added to wells of a 96‐well microplate pre‐coated with purified GPX4; 100 µL of biotinylated‐antibody working solution was also added to each well. After incubation, the liquid was discarded, and the plate was blotted dry. The wells were washed three times (200 µL per well, 2 min per wash) and blotted dry again. Next, 100 µL of HRP‐conjugated avidin working solution was added to each well, the plate was sealed with a new adhesive strip and incubated at 37°C for 1 h. Subsequent steps were performed exactly as described in the kit protocol. Finally, after addition of the stop solution, the absorbance at 450 nm was measured with a microplate reader.

### 4.5 Cellular Thermal Shift Assay

22RV1 cells were incubated for 24 h with the mixture prepared by co‐extruding binder 1–5 (25 µg/ml) and 22RV1 cell membrane‐derived vesicles (100 µg/ml). After harvesting, cells were washed twice with ice‐cold PBS (1000 rpm, 5 min), resuspended in PBS containing 1% (v/v) PMSF, and subjected to three freeze‐thaw cycles in liquid nitrogen (≈5 min each) to generate lysates. The cleared lysate was aliquoted into six 1.5 mL tubes and heated for 3 min at 58, 60, 62, and 64°C. Aggregated proteins were removed by centrifugation (14 000 rpm, 20 min, 4°C). Supernatants were mixed with SDS loading buffer, boiled for 15 min, and analyzed by Western blot; band intensities were quantified by densitometry.

### 4.6 Surface Plasmon Resonance Assays

SPR binding assays were performed on a Biacore 1K optical biosensor (Cytiva) at 25°C to characterize the interaction between recombinant GPX4 and the de novo‐designed peptides. A CM5 sensor chip was employed for all measurements. For ligand immobilization, the carboxymethyl dextran surface was activated by injecting a 1:1 (v/v) mixture of 0.4 M EDC and 0.1 M NHS at 10 µL/min for 420 s in 1.0 × PBS‐P+ (pH 7.4). Recombinant GPX4, diluted to 50 µg/mL in 10 mM sodium acetate (pH 4.0), was then covalently coupled to the activated surface via free primary amines under identical flow conditions. Residual active esters were subsequently quenched with 1 M ethanolamine hydrochloride (pH 8.5) at 10 µL/min for 420 s. A reference flow cell was prepared in parallel using the same activation and quenching protocol, except that the coupling step employed protein‐free 10 mM sodium acetate (pH 4.0) instead of the ligand solution, thereby enabling background subtraction to eliminate non‐specific binding and bulk refractive index effects. Kinetic analyses were conducted in 1.0 × PBS‐P+ (pH 7.4) containing 5% (v/v) DMSO as the running buffer. The peptides were serially diluted in PBST to generate a concentration gradient ranging from 1280 to 1.25 nM. Each concentration was injected over both the active and reference surfaces at 10 µL/min, with an association time of 100 s and a dissociation time of 120 s. Following each cycle, the sensor surface was regenerated by flowing 10 mM glycine‐HCl (pH 2.0) for 5 min to fully dissociate the bound analyte without compromising ligand integrity. The resulting sensorgrams were reference‐subtracted and globally fitted to a 1:1 Langmuir binding model to determine the association rate constant (ka), dissociation rate constant (kd), and equilibrium dissociation constant (KD).

### 4.7 Plasmid Construction

Full‐length human D1 and P1 open reading frames (ORFs) were inserted between the NheI and AsiSI sites of the pLVX backbone (no tag; ampicillin resistance, 100 µg mL^−^^1^). Correct insertion was confirmed by full‐length next‐generation sequencing. Glycerol stocks (500 µL) and purified plasmid DNA (50 µg, 100 – 200 ng µL^−^^1^) were shipped at ambient temperature and stored at −80°C (bacteria) or −20°C (plasmid) upon arrival.

For routine use, glycerol stocks were streaked onto LB‐agar plates containing 100 µg mL^−^^1^ ampicillin and incubated overnight at 37°C. Three single colonies were picked into 3 mL LB‐ampicillin liquid medium, grown overnight at 37°C with shaking (220 rpm), and plasmid DNA was isolated using a commercial kit (G3645‐10T, Servicebio, China). All preparations were sequence‐verified with the provided primers (5’‐end: CGCAAATGGGCGGTAGGCGTG; 3’‐end: CGTTCAATTGCCGACCCCTC) before downstream applications.

### 4.8 Cell Culture

Human 22RV1 prostate cancer cells and PC3 prostate cancer cells were purchased from Procell Life Science & Technology Co., Ltd. (Wuhan, Hubei, China). Both cell lines were authenticated by short‐tandem‐repeat (STR) profiling. All cells were confirmed mycoplasma‐negative by PCR and ensured absence of contamination by bacteria or mycoplasma. Complete RPMI‐1640 medium (KeyGEN, China) (supplemented with 10% fetal bovine serum (ABW, China, AB‐ FBS‐1050), 100 U/mL penicillin G, and 100 U/mL streptomycin sulfate) was prepared for routine culture of 22RV1 cells. Complete F‐12K medium (containing 10% fetal bovine serum, 100 U/mL penicillin G, and 100 U/mL streptomycin sulfate) was used for PC3 cells. Cell culture was performed at 37°C under 5% CO_2_ conditions in a humidified incubator.

### 4.9 Cell Transfection

22RV1 and PC3 cells were seeded into 6‐well plates at a density of 3 × 10^5^ cells per well and cultured for 12 h to allow attachment. Transfection was performed using the Lipofectamine (Lipo8000 Transfection Reagent, Beyotime, China) according to the manufacturer's protocol. For each well, 2.5 µg of plasmid and 4 µL of Lipo8000 were diluted in 125 µl of serum free medium, mixed gently, and incubated at room temperature for 30 min to form transfection complexes. The mixture was then added dropwise to the cells. After 24 h of transfection, the expression of GFP was observed under a fluorescence microscope to verify transfection efficiency.

### 4.10 Ubiquitination Assay

A degron‐mutant control sequence (‐RFGLGEER) was constructed by scrambling the identical composition to abrogate degron‐specific information, followed by fusion with the GPX4‐binder to generate bdC^m^. To determine whether bdC promotes GPX4 ubiquitination, cells were pretreated with MG132 (5 µM, 6 h) to halt proteasomal clearance and thereby accumulate ubiquitinated intermediates. Cells were then solubilized in a stringent lysis buffer supplemented with N‐ethylmaleimide to quench deubiquitinating enzymes. For GPX4 immunocapture, GPX4 antibody (0.2–2 µg; MCE, YA5931) was first incubated with magnetic beads (Co‐IP Kit, Abbkine) on a vertical rotator at room temperature for 30 min to generate antibody‐conjugated beads. Clarified extracts (0.2–1 mg total protein) from control, bdC‐, and bdCm‐treated cells were subsequently incubated with the antibody‐bead complexes on a vertical rotator at room temperature for 1 h. Following rigorous washing to strip away non‐covalent interactors, the immunoprecipitates were resolved by SDS‐PAGE and immunoblotted for ubiquitin conjugates (Abclonal, A23483). Whole‐cell lysates were probed in parallel to verify input levels, and IgG isotype controls were included to exclude non‐specific bead adsorption.

### 4.11 Quantitative Proteomic Analysis

Protein lysates were prepared in 8 M urea, quantified by BCA assay, and subjected to in‐solution digestion with trypsin (Trypsin/P, 37°C, 16 h) following reduction with DTT and alkylation with iodoacetamide. The resulting peptides were desalted on C18 StageTips, dried by vacuum centrifugation, and reconstituted in 0.1% formic acid. For each run, 150 ng of peptide material was separated on a Thermo Scientific Vanquish Neo UHPLC system equipped with an EASY‐Spray ES906 column (150 mm) using a 3.9 min linear gradient (mobile phase A: 0.1% formic acid in water; mobile phase B: 0.1% formic acid in 80% acetonitrile) at a flow rate of 3.0 µL/min. Eluted peptides were analyzed on an Orbitrap Astral mass spectrometer operating in positive‐ion DIA mode. Full‐scan MS1 spectra were acquired in the Orbitrap at 240 000 resolution (scan range m/z 380–980), followed by MS2 acquisition in 300 consecutive isolation windows (2 Th each) with HCD fragmentation at 25% normalized collision energy. DIA‐NN (v1.9.2) was employed for direct library‐free database searching (DirectDIA) against the human UniProt FASTA database. Search parameters included carbamidomethylation of cysteine as a fixed modification and oxidation of methionine plus protein N‐terminal acetylation as variable modifications, with up to two missed tryptic cleavages permitted. Peptide spectrum match and protein‐level false discovery rates were controlled at < 1% using a target‐decoy strategy.

### 4.12 Cell Membrane Derivative Isolation and Nanoparticle Preparation

Approximately 3–5 × 10^7^ cells were harvested, washed twice with sterile PBS, detached with 0.25% trypsin‐EDTA, neutralized with complete medium, and collected by centrifugation. Two additional washes with sterile PBS followed. The pellet was resuspended in 1 mL sterile PBS and lysed by 5 freeze–thaw cycle. The lysate was then sonicated on ice for 1 min (3s on/3s off, 10% amplitude) to fragment cellular membranes. The homogenate was centrifuged at 700 g, 4°C, 10 min to remove nuclei and intact cells; the supernatant was transferred to ultracentrifuge tubes and centrifuged at 14 000 g, 4°C, 60 min to pellet membrane fragments. The membrane‐rich pellet was resuspended in 1 mL sterile PBS and sequentially extruded through a 200 nm polycarbonate membrane in a LiposoFast handheld lipid extruder (Avestin) for 50 cycles at 22°C to generate homogeneous cell membrane‐derived nanoparticles.

### 4.13 Flow Cytometric Analysis of Ferroptosis

Following transfection, cells were harvested to evaluate ferroptosis levels. Intracellular ferrous iron (Fe^2+^) was detected using the Cellular Red Fluorescent Ferrous Ion Assay Kit with RhoNox‐6 (Beyotime, China), and reactive oxygen species (ROS) were assessed using the ROS Assay Kit for Superoxide Anion with DHE (Beyotime, China). Briefly, the transfected cells were collected and incubated with RhoNox‐6 working solution or DHE probe at 37°C for 30 min in the dark in parallel samples. After washing with PBS, fluorescence intensity was measured using a flow cytometer Neon‐1026 M (BEAMDIAG, China). Data analysis was performed using FlowJo v10.9.0.

### 4.14 Click Chemistry

25 µM Ac4ManNAz (HY‐W728531, MedChemExpress, China) was mixed with RPMI 1640 medium supplemented with 10% FBS and 1% penicillin‐streptomycin and co‐cultured for 72 h, allowing Ac4ManNAz to be incorporated into the glycoproteins on the surface of live 22RV1 cells. 1 × 10^5^ 22RV1 cells were seeded into confocal dishes and maintained in medium containing Ac4ManNAz. Subsequently, the cells were incubated with 10 µM DBCO‐PSMAapt‐Cy3 (synthesized by Sangon Biotech) for 1 h, during which a click reaction occurred between DBCO and Ac4ManNAz. The occurrence of the click reaction was verified using confocal microscopy. Cells were fixed with 4% paraformaldehyde for 20 min, stained with phalloidin 488 for 30 min to visualize the cytoskeleton, and nuclei were stained with DAPI for 10–20 min as required. Confocal microscopy was used to observe the localization of Cy3 red fluorescence on the cell membrane.

### 4.15 Agarose Gel Retardation Assays

Samples containing naked plasmid or vesicle‐encapsulated plasmid were loaded on 1% agarose gels, run at 120 V for 45 min, stained with Goldview (G3073‐1ML, Servicebio, China) and imaged by ChemiDoc MP (Bio‐Rad, USA).

### 4.16 Colony Formation Assay

For the colony formation assay, transfected 22RV1 and PC3 cells were digested and seeded into 6‐well plates at a density of 1000 cells per well. The cells were cultured in complete medium at 37°C with 5% CO_2_ for 10–14 days to allow colony formation, with the medium replaced every 3 days. Upon the appearance of macroscopic colonies, the medium was discarded, and the cells were washed twice with PBS. The colonies were then fixed with 4% paraformaldehyde for 30 min and stained with 0.1% crystal violet solution for 30 min at room temperature. After washing away the excess dye and air‐drying, the plates were photographed.

### 4.17 Transwell Migration Assay

Drug‐treated and control 22RV1 cells were starved overnight, harvested, and resuspended in serum‐free medium. 8 × 10^4^ cells in 200 µL of serum‐free medium were seeded into the upper compartment of each 8‐µm‐pore Transwell insert, and 600 µL of medium containing 10% FBS was added to the lower well as a chemoattractant. Following 48 h incubation at 37°C, 5% CO_2_, non‐migrated cells on the upper membrane surface were carefully removed with a cotton swab. The inserts were washed twice with PBS, fixed with 4% paraformaldehyde for 20 min, and stained with 0.1% crystal violet for 15 min. Excess dye was rinsed off with PBS, and migrated cells adhering to the underside of the membrane were retained for imaging and quantification.

### 4.18 Fluorescent and Bioluminescence Imaging in Vivo

The in vivo imaging was performed using two distinct modalities to evaluate biodistribution and therapeutic efficacy. For the biodistribution analysis, nanoparticles were labeled with the near‐infrared dye DiR. BALB/c nude mice were intravenously injected via the tail vein with the DiR‐labeled vesicles (100 µl per mouse). The concentration of the nanoparticles was adjusted to 20 µg/ml based on a BCA assay prior to injection. Fluorescence imaging was conducted using the AniView Phoenix X system (BLT, China) with excitation and emission filters specific for DiR at 1, 6, 12, 24, 48, and 72 h post‐injection. For the evaluation of therapeutic efficacy, a bioluminescence imaging model was employed using luciferase‐expressing 22RV1 cells. To monitor tumor growth, mice were intraperitoneally injected with D‐luciferin potassium salt (1.5 mg dissolved in 150 µl PBS per mouse). Images were acquired 5 min after substrate injection using the AniView Phoenix X system. Imaging was performed every 3 days to track the bioluminescence intensity, which correlates with tumor burden.

### 4.19 In Vivo Antitumor Efficacy

The Animal Ethics Committee of Tongji Hospital, Huazhong University of Science and Technology, granted approval for all animal studies under protocol number TJH‐202504022. All procedures were performed in strict compliance with institutional regulations. Male BALB/c nude mice (6 weeks old) were purchased and housed under specific pathogen‐free (SPF) conditions. To establish the tumor xenograft model, 5 × 10^6^ luciferase‐expressing 22RV1 cells suspended in 100 µL of PBS (mixed with Matrigel, 1:1) were subcutaneously injected into the right flank of each mouse. When the average tumor volume reached approximately 100mm^3^, the mice were randomly divided into 6 groups (n = 4 per group). The mice were then treated with PBS or different formulations via tail vein injection every 3 days. During the treatment period, tumor volume (Vt) and body weight were monitored every 3 days. Tumor volume was calculated using the formula (Vt = length × width^2^)/2. At the end of the experiment, blood samples were collected from the retro‐orbital sinus of all mice. The serum was separated by centrifugation to evaluate liver and kidney functions (including ALT, AST, BUN, and CRE) using an automatic biochemical analyzer. Additionally, serum malondialdehyde (MDA) levels were measured to assess oxidative stress. Subsequently, all mice were sacrificed. The tumors were excised, weighed, photographed, and preserved for further histological analysis.

### 4.20 Immunohistochemistry

Tumor tissues were fixed in 4% paraformaldehyde, embedded in paraffin, and sectioned at a thickness of 4 µm. After deparaffinization and rehydration, antigen retrieval was performed in citrate buffer (pH 6.0) at boiling temperature. The sections were blocked with 5% BSA (or goat serum) and incubated with the anti‐GPX4 primary antibody (1:100 dilution, Abmart, China) overnight at 4°C. Subsequently, the sections were incubated with an HRP‐conjugated secondary antibody, followed by staining with a DAB substrate kit. Nuclei were counterstained with hematoxylin. Images were captured using a light microscope.

### 4.21 Hemolytic Assay

Whole blood (1 mL) was withdrawn from the retro‐orbital sinus of anesthetized BALB/c nude mice into heparinized tubes and centrifuged immediately at 3 000 rpm (1000 g, 10 min, 4°C). The erythrocyte pellet was washed three times with ice‐cold sterile PBS (pH 7.4) and re‐suspended to 4% (v/v). Test materials (0.5 mL, serially diluted in PBS) were mixed with 0.5 mL of the cell suspension and incubated at 37°C for 1 h with gentle shaking. After a second centrifugation (3000 rpm, 5 min), supernatant absorbance was measured at 540 nm. PBS and 0.1% Triton X‐100 served as 0% and 100% hemolysis controls; hemolysis (%) = [(A540 sample − A540 PBS)/ (A540 Triton − A540 PBS)] × 100.

### 4.22 Data Analysis and Mapping

Results are reported as mean ± SD. Statistical evaluations were carried out with GraphPad Prism 8.0. Comparisons between two groups were analyzed by unpaired two‐tailed Student's t‐test. For comparisons among multiple groups, one‐way analysis of variance (ANOVA) was employed, followed by Tukey's post‐hoc test for multiple pairwise comparisons. A p‐value < 0.05 was considered statistically significant (^*^ *p* < 0.05, ^**^ *p* < 0.01, ^***^ *p* < 0.001, ^****^ *p* < 0.0001).

## Author Contributions

S.H.Z. conceived the project. S.H.Z., Y.A., N.Z., M.C.L., and J.X.S. designed experiments. S.H.Z., Y.A., N.Z., and X.G. collected data. J.Z.X., Q.D.X., and J.X.S. gave technical advice. S.H.Z., N.Z., M.C.L., and X.G. performed data analysis and interpreted results. S.H.Z., Y.A., J.Z.X., and J.X.S. created figures. S.H.Z., Y.A., and N.Z. wrote the initial manuscript draft. S.G.W., Q.D.X., J.X.S., and J.Z.X. supervised the project. S.G.W., Q.D.X., and J.X.S. provided funding for the project. All authors reviewed the final manuscript.

## Funding

This work was financially supported by the Natural Science Foundation of Hubei Funding Province (Grant No. 2022CFB477), the Tongji Hospital Medical Innovation and Transformation Incubation Project (Grant No. 2022ZHF Y02), the China Postdoctoral Science Foundation (GrantNo. 2024M761035), the Postdoctoral Research Startup Fund of Tongji Hospital (Grant No. 24‐2RSC09043‐32), the Tongji Hospital Fund Incubation Program (Grant No. 2025B11), and the National Key Research and Development Program of China: Research on Precision Subtyping and Stratified Treatment Optimization of Prostate Cancer Based on Clinical Multi‐omics Big Data (Grant No. 2026ZD0553300).

## Ethics Statement

All animal experimental procedures were carried out in compliance with the ARRIVE guidelines and the National Institutes of Health guide for the care and use of Laboratory animals. The study protocol was approved by the Animal Care and Use Committee of Tongji Hospital, Huazhong University of Science and Technology (Approval No.: TJH‐202504022).

## Conflicts of Interest

The authors declare no conflicts of interest.

## Supporting information




**Supporting File**: advs77932‐sup‐0001‐SuppMat.docx.

## Data Availability

The data that support the findings of this study are available from the corresponding author upon reasonable request.

## References

[1] F. Bray , M. Laversanne , H. Sung , et al., “Global Cancer Statistics 2022: GLOBOCAN Estimates of Incidence and Mortality Worldwide for 36 Cancers in 185 Countries,” CA: A Cancer Journal for Clinicians 74 (2024): 229–263. 10.3322/caac.21834 38572751

[2] T. Chandrasekar and D. Tilki , “Comparing Quality of Life Outcomes After Prostate Cancer Treatment,” Nature Reviews Urology 14, no. 7 (2017): 396–397, 10.1038/nrurol.2017.81. 28607497

[3] P. H. A. Cao , A. Dominic , F. E. Lujan , et al., “Unlocking Ferroptosis in Prostate Cancer‐The Road to Novel Therapies and Imaging Markers,” Nature Reviews Urology 21, no. 10 (2024): 615–637, 10.1038/s41585-024-00869-9. PMC12067944 38627553

[4] W. S. Yang , R. SriRamaratnam , M. E. Welsch , et al., “Regulation of Ferroptotic Cancer Cell Death by GPX4,” Cell 156, no. 1‐2 (2014): 317–331, 10.1016/j.cell.2013.12.010. PMC4076414 24439385

[5] J. K. Eaton , L. Furst , R. A. Ruberto , et al., “Selective Covalent Targeting of GPX4 Using Masked Nitrile‐Oxide Electrophiles,” Nature Chemical Biology 16, no. 5 (2020): 497–506, 10.1038/s41589-020-0501-5. PMC7251976 32231343

[6] J. L. Watson , D. Juergens , N. R. Bennett , et al., “De Novo Design of Protein Structure and Function With RFdiffusion,” Nature 620, no. 7976 (2023): 1089–1100, 10.1038/s41586-023-06415-8. PMC10468394 37433327

[7] Y. Yan , H. Tao , J. He , and S.‐Y. Huang , “The HDOCK Server for Integrated Protein–Protein Docking,” Nature Protocols 15, no. 5 (2020): 1829–1852, 10.1038/s41596-020-0312-x. 32269383

[8] M. Pacesa , L. Nickel , C. Schellhaas , et al., “One‐Shot Design of Functional Protein Binders With BindCraft,” Nature 646, no. 8084 (2025): 483–492, 10.1038/s41586-025-09429-6. PMC12507698 40866699

[9] X. Wu , L.‐Y. Xu , E.‐M. Li , and G. Dong , “Application of Molecular Dynamics Simulation in Biomedicine,” Chemical Biology & Drug Design 99, no. 5 (2022): 789–800, 10.1111/cbdd.14038. 35293126

[10] M. Békés , D. R. Langley , and C. M. Crews , “PROTAC Targeted Protein Degraders: The Past Is Prologue,” Nature Reviews Drug Discovery 21 (2022): 181–200. 10.1038/s41573-021-00371-6 PMC8765495 35042991

[11] L. B. Snyder , T. K. Neklesa , R. R. Willard , et al., “Preclinical Evaluation of Bavdegalutamide (ARV‐110), a Novel PROteolysis TArgeting Chimera Androgen Receptor Degrader,” Molecular Cancer Therapeutics 24, no. 4 (2025): 511–522, 10.1158/1535-7163.MCT-23-0655. PMC11962395 39670468

[12] Z. Li , C. Zhu , Y. Ding , Y. Fei , and B. Lu , “ATTEC: A Potential New Approach to Target Proteinopathies,” Autophagy 16, no. 1 (2020): 185–187, 10.1080/15548627.2019.1688556. PMC6984452 31690177

[13] S. An and L. Fu , “Small‐Molecule PROTACs: An Emerging and Promising Approach for the Development of Targeted Therapy Drugs,” EBioMedicine 36 (2018): 553–562. 10.1016/j.ebiom.2018.09.005 PMC6197674 30224312

[14] S. Ma , J. Ji , Y. Tong , et al., “Non‐Small Molecule PROTACs (NSM‐PROTACs): Protein Degradation Kaleidoscope,” Acta pharmaceutica Sinica B 12 (2022): 2990–3005. 10.1016/j.apsb.2022.02.022 PMC9293674 35865099

[15] D. Zhang , B. Ma , D. Liu , et al., “Discovery of a Peptide Proteolysis‐Targeting Chimera (PROTAC) Drug of p300 for Prostate Cancer Therapy,” eBioMedicine 105 (2024): 105212. 10.1016/j.ebiom.2024.105212 PMC11261775 38954976

[16] A. Varshavsky , “N‐Degron Pathways,” Proceedings of the National Academy of Sciences 121, no. 39 (2024): 2408697121, 10.1073/pnas.2408697121. PMC11441550 39264755

[17] Y. Makaros , A. Raiff , R. T. Timms , et al., “Ubiquitin‐Independent Proteasomal Degradation Driven by C‐Degron Pathways,” Molecular Cell 83, no. 11 (2023): 1921–1935, 10.1016/j.molcel.2023.04.023. PMC10237035 37201526

[18] M. S. Kim , H. K. Bhargava , G. E. Shavey , W. A. Lim , H. El‐Samad , and A. H. Ng , “Degron‐Based bioPROTACs for Controlling Signaling in CAR T Cells,” ACS Synthetic Biology 13, no. 8 (2024): 2313–2327, 10.1021/acssynbio.4c00109. PMC11334183 38991546

[19] Z. Zhang , B. Sie , A. Chang , et al., “Elucidation of E3 Ubiquitin Ligase Specificity Through Proteome‐Wide Internal Degron Mapping,” Molecular Cell 83, no. 18 (2023): 3377–3392, 10.1016/j.molcel.2023.08.022. PMC10594193 37738965

[20] E. Blanco , H. Shen , and M. Ferrari , “Principles of Nanoparticle Design for Overcoming Biological Barriers to Drug Delivery,” Nature Biotechnology 33, no. 9 (2015): 941–951, 10.1038/nbt.3330. PMC4978509 26348965

[21] D. Winkelvoß , D. Vukovic , A. C. Hänny , et al., “Molecular Features Defining the Efficiency of bioPROTACs,” Communications Biology 8 (2025): 946. 10.1038/s42003-025-08352-w PMC12181330 40542219

[22] R. Zhou , H. Wang , G.‐M. Zhang , et al., “Targeted Degradation of Endogenous YAP by Nanobody bioPROTAC Inhibits Tumor Progression,” Nature Communications 16, no. 1 (2025): 9374, 10.1038/s41467-025-64426-7. PMC12549912 41130953

[23] S. Lim , R. Khoo , K. M. Peh , et al., “bioPROTACs as Versatile Modulators of Intracellular Therapeutic Targets Including Proliferating Cell Nuclear Antigen (PCNA),” Proceedings of the National Academy of Sciences 117, no. 11 (2020): 5791–5800, 10.1073/pnas.1920251117. PMC7084165 32123106

[24] L. Taiariol , C. Chaix , C. Farre , and E. Moreau , “Click and Bioorthogonal Chemistry: The Future of Active Targeting of Nanoparticles for Nanomedicines?,” Chemical Reviews 122, no. 1 (2022): 340–384, 10.1021/acs.chemrev.1c00484. 34705429

[25] P. L. Rodriguez , T. Harada , D. A. Christian , D. A. Pantano , R. K. Tsai , and D. E. Discher , “Minimal "Self" Peptides That Inhibit Phagocytic Clearance and Enhance Delivery of Nanoparticles,” Science 339, no. 6122 (2013): 971–975, 10.1126/science.1229568. PMC3966479 23430657

[26] Y. Xie , R. Kang , D. J. Klionsky , and D. Tang , “GPX4 in Cell Death, Autophagy, and Disease,” Autophagy 19, no. 10 (2023): 2621–2638, 10.1080/15548627.2023.2218764. PMC10472888 37272058

[27] T. Tsujino , S. Yamazaki , M. Sakamoto , et al., “A Ferroptosis‐Suppressive state Drives Resistance to Bladder‐Preserving Chemoradiotherapy in Muscle‐Invasive Bladder Cancer,” Signal Transduction and Targeted Therapy 11, no. 1 (2026): 299, 10.1038/s41392-026-02847-6. PMC13379577 42469227

[28] D. Moosmayer , A. Hilpmann , J. Hoffmann , et al., “Crystal Structures of the Selenoprotein Glutathione Peroxidase 4 in Its Apo Form and in Complex With the Covalently Bound Inhibitor ML162,” Biological Crystallography 77 (2021): 237–248. 10.1107/S2059798320016125 PMC7869902 33559612

[29] H. Liu , F. Forouhar , A. J. Lin , et al., “Small‐Molecule Allosteric Inhibitors of GPX4,” Cell Chemical Biology 29, no. 12 (2022): 1680–1693, 10.1016/j.chembiol.2022.11.003. PMC9772252 36423641

[30] G. P. Pinto , N. M. Hendrikse , J. Stourac , J. Damborsky , and D. Bednar , “Virtual Screening of Potential Anticancer Drugs Based on Microbial Products,” Seminars in Cancer Biology 86 (2022): 1207–1217, 10.1016/j.semcancer.2021.07.012. 34298109

[31] F. Gentile , J. C. Yaacoub , J. Gleave , et al., “Artificial Intelligence–Enabled Virtual Screening of Ultra‐Large Chemical Libraries With Deep Docking,” Nature Protocols 17, no. 3 (2022): 672–697, 10.1038/s41596-021-00659-2. 35121854

[32] H. Jiang , J. Wang , W. Cong , et al., “Predicting Protein–Ligand Docking Structure With Graph Neural Network,” Journal of Chemical Information and Modeling 62, no. 12 (2022): 2923–2932, 10.1021/acs.jcim.2c00127. PMC10279412 35699430

[33] M. Varadi , S. Anyango , M. Deshpande , et al., “AlphaFold Protein Structure Database: Massively Expanding the Structural Coverage of Protein‐Sequence Space With High‐Accuracy Models,” Nucleic Acids Research 50, no. D1 (2022): D439–D444, 10.1093/nar/gkab1061. PMC8728224 34791371

[34] C. Zheng , C. Wang , D. Sun , et al., “Structure‐Activity Relationship Study of RSL3‐based GPX4 Degraders and Its Potential Noncovalent Optimization,” European Journal of Medicinal Chemistry 255 (2023): 115393, 10.1016/j.ejmech.2023.115393. 37098297

[35] C. Wang , C. Zheng , H. Wang , et al., “Dual Degradation Mechanism of GPX4 Degrader in Induction of Ferroptosis Exerting Anti‐resistant Tumor Effect,” European Journal of Medicinal Chemistry 247 (2023): 115072, 10.1016/j.ejmech.2022.115072. 36603510

[36] M. Schneider , C. J. Radoux , A. Hercules , et al., “The PROTACtable Genome,” Nature Reviews Drug Discovery 20, no. 10 (2021): 789–797, 10.1038/s41573-021-00245-x. 34285415

[37] A. Varshavsky , “N‐Degron and C‐Degron Pathways of Protein Degradation,” Proceedings of the National Academy of Sciences U S A 116, no. 2 (2019): 358–366, 10.1073/pnas.1816596116. PMC6329975 30622213

[38] I. Koren , R. T. Timms , T. Kula , Q. Xu , M. Z. Li , and S. J. Elledge , “The Eukaryotic Proteome Is Shaped by E3 Ubiquitin Ligases Targeting C‐Terminal Degrons,” Cell 173, no. 7 (2018): 1622–1635, 10.1016/j.cell.2018.04.028. PMC6003881 29779948

[39] J. Qu , X. Ren , F. Xue , et al., “Specific Knockdown of α‐Synuclein by Peptide‐Directed Proteasome Degradation Rescued Its Associated Neurotoxicity,” Cell Chemical Biology 27, no. 6 (2020): 751–762, 10.1016/j.chembiol.2020.03.010. 32359427

[40] F. Liu , J. Chen , K. Li , et al., “Ubiquitination and Deubiquitination in Cancer: From Mechanisms to Novel Therapeutic Approaches,” Molecular Cancer 23, no. 1 (2024): 148, 10.1186/s12943-024-02046-3. PMC11270804 39048965

[41] A. Fletcher , D. Clift , E. de Vries , et al., “A TRIM21‐Based bioPROTAC Highlights the Therapeutic Benefit of HuR Degradation,” Nature Communications 14, no. 1 (2023): 7093, 10.1038/s41467-023-42546-2. PMC10625600 37925433

[42] N. Pardi and F. Krammer , “mRNA Vaccines for Infectious Diseases‐Advances, Challenges and Opportunities,” Nature reviews Drug discovery 23 (2024): 838–861. 10.1038/s41573-024-01042-y PMC13101425 39367276

[43] Z. Li , G. Qiao , X. Wang , et al., “De Novo Designed Protein Guiding Targeted Protein Degradation,” Nature Communications 16, no. 1 (2025): 6598, 10.1038/s41467-025-62050-z. PMC12271464 40676011

[44] A. Chan , R. M. Haley , M. A. Najar , et al., “Lipid‐Mediated Intracellular Delivery of Recombinant bioPROTACs for the Rapid Degradation of Undruggable Proteins,” Nature Communications 15, no. 1 (2024): 5808, 10.1038/s41467-024-50235-x. PMC11237011 38987546

[45] N.‐Y. Zhang , D.‐Y. Hou , X.‐J. Hu , et al., “Nano Proteolysis Targeting Chimeras (PROTACs) With Anti‐Hook Effect for Tumor Therapy,” Angewandte Chemie International Edition 62, no. 37 (2023): 202308049, 10.1002/anie.202308049. 37486792

[46] W. Du , C. Chen , Y. Liu , et al., “A Combined “Eat Me/Don't Eat Me” Strategy Based on Exosome for Acute Liver Injury Treatment,” Cell Reports Medicine 6, no. 4 (2025): 102033, 10.1016/j.xcrm.2025.102033. PMC12047510 40120577

[47] J. Deng , S. Zhao , J. Li , et al., “One‐Step Thermophoretic AND Gate Operation on Extracellular Vesicles Improves Diagnosis of Prostate Cancer,” Angewandte Chemie International Edition 61, no. 33 (2022): 202207037, 10.1002/anie.202207037. 35749531

[48] T. Smyth , K. Petrova , N. M. Payton , et al., “Surface Functionalization of Exosomes Using Click Chemistry,” Bioconjugate Chemistry 25, no. 10 (2014): 1777–1784, 10.1021/bc500291r. PMC4198107 25220352

[49] A. Lamoot , A. Uvyn , S. Kasmi , and B. G. De Geest , “Covalent Cell Surface Conjugation of Nanoparticles by a Combination of Metabolic Labeling and Click Chemistry,” Angewandte Chemie International Edition 60, no. 12 (2021): 6320–6325, 10.1002/anie.202015625. 33368900

[50] L. Wang , “Engineering the Genetic Code in Cells and Animals: Biological Considerations and Impacts,” Accounts of Chemical Research 50, no. 11 (2017): 2767–2775, 10.1021/acs.accounts.7b00376. PMC5698093 28984438

[51] H.‐T. Huang , R. J. Lumpkin , R. W. Tsai , et al., “Ubiquitin‐Specific Proximity Labeling for the Identification of E3 Ligase Substrates,” Nature Chemical Biology 20, no. 9 (2024): 1227–1236, 10.1038/s41589-024-01590-9. 38514884

[52] K. Newton , A. Strasser , N. Kayagaki , and V. M. Dixit , “Cell Death,” Cell 187 (2024): 235–256. 10.1016/j.cell.2023.11.044 38242081

