# Supplementary material for: Degrading GPX4 via Biomimetic Nanoparticle‐Mediated In Situ Synthesis of Deep‐Learning‐Designed Binder‐Degron Chimeras for Prostate Cancer Therapy
Source: Adv Sci (Weinh). 2026 Sep 27:e77932. Online ahead of print. doi: 10.1002/advs.77932 (PMC13616326; doi:10.1002/advs.77932)
Supplement: Supplementary file 1 [file ADVS-9999-e77932-s001.docx]

**Supplementary Materials**

**Title: Degrading GPX4 via Biomimetic Nanoparticle-Mediated in Situ Synthesis of Deep-Learning-Designed Binder-Degron Chimeras for Prostate Cancer Therapy**

**Author names and affiliations:**

Si-Han Zhang^1*^, Ye An^2*^, Na Zeng^1*^, Mei-Cheng Liu^1^, Jian-Xuan Sun^1^, Xi Gong^1^, Jin-Zhou Xu^1^, Shao-Gang Wang^1#^, Qi-Dong Xia^1 #^

1. Department and Institute of Urology, Tongji Hospital, Tongji Medical College, Huazhong University of Science and Technology, No. 1095 Jiefang Avenue, 430030, Wuhan, China.
2. Department of Urology, The First People's Hospital of Yunnan Province, Kunming University of Science and Technology Affiliated Hospital, Kunming, China

*: These three authors contributed equally to this work.

#: These two corresponding authors contributed equally to this work.

**Corresponding author:**

Qi-Dong Xia, Department and Institute of Urology, Tongji Hospital, Tongji Medical College, Huazhong University of Science and Technology, Wuhan, P.R. China. E-mail: qidongxia_md@163.com Telephone numbers: +86-027-83663460

Shao-Gang Wang, Department and Institute of Urology, Tongji Hospital, Tongji Medical College, Huazhong University of Science and Technology, Wuhan, P.R. China. E-mail: sgwangtjm@163.com Telephone numbers: Tel. +86-027-83663460.

This file contains Supplementary Figures 1-22 and Supplementary Tables 1.

**Supplementary Figure 1**: Docking diagrams of the top 10 binders generated by Bindcraft with GPX4 (PDB: 6ELW).

**Supplementary Figure 2**: Re-docking of GPX4 with the binder using H-DOCK. The top 10 binding models are shown with their respective docking scores.

| ID | Cavities_volume | center_x | center_y | center_z | contact_resi |
| --- | --- | --- | --- | --- | --- |
| 1 | 247 | -17.228 | 3.156 | 19.971 | 125:K:A,127:K:A,128:G:A,129:I:A,130:L:A,135:K:A,139:T:A,151:K:A,152:R:A,153:Y:A,154:G:A,155:P:A,156:M:A,157:E:A |
| 2 | 133 | -9.306 | -11.263 | 23.048 | 9:R:A,10:C:A,11:A:A,12:R:A,119:W:A,122:I:A,123:Q:A,124:P:A,125:K:A,148:C:A,149:V:A |
| 3 | 108 | -2.211 | -0.359 | 0.013 | 21:D:A,22:I:A,23:D:A,27:V:A,31:K:A,32:Y:A,90:K:A,93:A:A,94:A:A,98:V:A,100:F:A,101:D:A,102:M:A,103:F:A |
| 4 | 97 | -20.278 | 7.766 | 7.245 | 43:A:A,45:Q:A,47:G:A,48:K:A,49:T:A,52:N:A,81:Q:A,136:W:A,137:N:A,138:F:A,155:P:A,156:M:A |
| 5 | 94 | 2.78 | -1.557 | 21.276 | 34:G:A,35:F:A,36:V:A,66:S:A,67:G:A,144:D:A,145:K:A,146:N:A,168:H:A,169:Y:A,170:F:A,171:H:A |

**Supplementary Table 1:** By analyzing the GPX4 crystal structure with the CBdock2 cavity-searching function, we identified the five largest surface cavities (numbered 1–5) as potential protein–protein interaction sites; their volumes, geometric centers, bounding-box dimensions and lining residues are listed.

**Supplementary Figure 3**: Variations in Interface Predicted Aligned Error (i_pae), predicted TM-score (ptm) and Radius of Gyration (rg) during the generation process of Binder1-5.

**Supplementary Figure 4**: Molecular dynamics simulations of five GPX4-Binder complexes: Variations in Root Mean Square Deviation (RMSD), radius of gyration, and Solvent Accessible Surface Area (SASA) over 100 ns.

**Supplementary Figure 5**: The changes in the Root Mean Square Fluctuation (RMSF) values of chain A and chain B in the GPX4-Binder complex for different residues (chain A: GPX4, chain B: Binder).

**Supplementary Figure 6**: Ramachandran Plot: The x- and y-axes represent the phi and psi torsion angles, respectively. The blue region denotes the normal (allowed) area, while the red points indicate outliers. The transparency of the colors reflects the density or frequency of data points, with darker shades indicating higher density. The orange and green regions represent areas with lower data point density. (General: The overall Ramachandran plot for the protein; GLY: The Ramachandran plot for glycine residues; Pre-PRO: The distribution of dihedral angles for residues preceding proline; PRO: The distribution of dihedral angles for proline residues.)

**Supplementary Figure 7:** The radius of gyration of binder5 in a 100 ns molecular dynamics simulation varies in the x, y, and z directions.

**HPLC Analysis Report**

Sample ID: binder1

Sequence: Biotin-MEEIKKEIVELFEKLNTEKDPNDKEIMEKLGELYPKIM

Column: 4.6*250mm, kromasil C18-5

Solvent A: 0.1%Trifluoroacetic in 100% Acetonirile

Solvent B: 0.1%Trifluoroacetic in 100% Water

Gradient: A	B

0.01min	5%	95%

25.0min	95%	5%

30min	90%	10%

Flow rate: 1.0ml/min	Wavelength: 214nm	Volume: 20ul

**MS analysis report**

Sample ID: binder1

Expected MS: 4822.64

Flow rate: 0.2ml/min	Run Time: 1min

Buffer A: 0.1% HCOOH in water	Buffer B: 0.1% HCOOH in Acetonirile

**Supplementary Figure 8:** HPLC and MS analysis of the synthesized Binder 1.

**HPLC Analysis Report**

Sample ID: Binder2

Sequence: Biotin-SAEKEKELTELYNKIKEKKWTDPPQEEVDKVVKLMEEVWG

Column: 4.6*250mm, kromasil C18-5

Solvent A: 0.1%Trifluoroacetic in 100% Acetonirile

Solvent B: 0.1%Trifluoroacetic in 100% Water

Gradient: A	B

0.01min	5%	95%

25.0min	95%	5%

30min	90%	10%

Flow rate: 1.0ml/min	Wavelength: 214nm	Volume: 20ul

**MS Analysis Report**

Sample ID**:** Binder2

Expected MS**:** 5031.71

Flow rate**:** 0.2ml/min	Run Time**:** 1min

Buffer A**:** 0.1% HCOOH in water	Buffer B**:** 0.1% HCOOH in Acetonirile

**Supplementary Figure 9:** HPLC and MS analysis of the synthesized Binder 2.

**HPLC Analysis Report**

Sample ID**:** Binder3

Sequence**:** Biotin-DQDKKMEEFYYYVVKNKITDEEEIDKLYNEIMNS

Column**:** 4.6*250mm, kromasil C18-5

Solvent A**:** 0.1%Trifluoroacetic in 100% Acetonirile

Solvent B**:** 0.1%Trifluoroacetic in 100% Water

Gradient**:** A	B

0.01min	5%	95%

25.0min	95%	5%

30min	90%	10%

Flow rate**:** 1.0ml/min	Wavelength**:** 214nm	Volume**:** 20ul

**MS Analysis Report**

Sample ID: Binder3

Expected MS: 4491.98

Flow rate: 0.2ml/min	Run Time: 1min

Buffer A: 0.1% HCOOH in water	Buffer B: 0.1% HCOOH in Acetonirile

**Supplementary Figure 10:** HPLC and MS analysis of the synthesized Binder 3.

**HPLC Analysis Report**

Sample ID**:** Binder4

Sequence**:** Biotin-MKITEEEMDKYYHELKYTDPKKLQEIWDNNEIVN

Column**:** 4.6*250mm, kromasil C18-5

Solvent A**:** 0.1%Trifluoroacetic in 100% Acetonirile

Solvent B**:** 0.1%Trifluoroacetic in 100% Water

Gradient**:** A	B

0.01min	5%	95%

25.0min	95%	5%

30min	90%	10%

Flow rate**:** 1.0ml/min	Wavelength**:** 214nm	Volume**:** 20ul

**MS Analysis Report**

Sample ID**:** Binder4

Expected MS**:** 4515.06

Flow rate**:** 0.2ml/min	Run Time**:** 1min

Buffer A**:** 0.1% HCOOH in water	Buffer B**:** 0.1% HCOOH in Acetonirile

**Supplementary Figure 11:** HPLC and MS analysis of the synthesized Binder 4.

**HPLC Analysis Report**

Sample ID: Binder5

Sequence: Biotin-NENLEVSNKLHDLKDKDPEEYRKLYKEQFEKEGMTWMED

Column: 4.6*250mm, kromasil C18-5

Solvent A: 0.1%Trifluoroacetic in 100% Acetonirile

Solvent B: 0.1%Trifluoroacetic in 100% Water

Gradient: A	B

0.01min	5%	95%

25.0min	95%	5%

30min	90%	10%

Flow rate: 1.0ml/min	Wavelength: 214nm	Volume: 20ul

**MS Analysis Report**

Sample ID: Binder5

Expected MS: 5086.60

Flow rate: 0.2ml/min	Run Time: 1min

Buffer A: 0.1% HCOOH in water	Buffer B: 0.1% HCOOH in Acetonirile

**Supplementary Figure 12:** HPLC and MS analysis of the synthesized Binder 5.

**Supplementary Figure 13**: ELISA results showing binding efficacy of binders. Binder5 exhibited the highest binding efficacy at the same concentration, while binder4 also demonstrated good binding performance.

**Supplementary Figure 14**: 22RV1 cells were lysed by repeated freeze-thaw cycles and centrifuged to extract the cell membrane. Then, the GPX4 peptide based PROTAC (GPX4-p-PROTAC) labeled with FITC was co-extruded with the 22RV1 cell membrane using a 200 nm membrane. At the same peptide concentration, the GPX4-p-PROTAC loaded with extracellular vesicles (GPX4-p-PROTAC@EVs) showed a more diffuse distribution in the in vitro cultured 22RV1 cells (Scale bar, 50 μm).

**Supplementary Figure 15:** Construction of CD47-overexpressing 293T cells. Plasmid transfection was performed using the electroporation method (GEMINITM & ECM® 830 ELECTROPORATION PROTOCOL). CD47 overexpression plasmids (CD47-OE plasmids, pLV3-CMV-Cd47(mouse)-3xFLAGCopGFP-Puro), Control plasmids (pLV3-CMV-3xFLAGCopGFP-Puro), Scale bar, 50 μm.

**Supplementary Figure 16**: Structural generation and molecular docking analysis of the PSMA-targeting aptamer. (**A**) Secondary structure prediction of the aptamer performed using SnapGene. The most stable conformation with the lowest minimum free energy (ΔG=-5.10 kcal/mol) is displayed on the left, alongside alternative folding patterns. The color gradient (red to blue) indicates the reliability of the base-pairing probabilities. (**B**) Three-dimensional (3D) tertiary structure modeling of the aptamer generated by the 3dDNA server. Five representative conformations are shown with their corresponding 3dDNA scores. (**C**) Superimposition of the top five 3D models predicted by 3dDNA (left) and AlphaFold3 (right). (**D**) Molecular docking simulation revealing the potential binding mode between the aptamer and the Prostate-Specific Membrane Antigen (PSMA) protein. The surface representation (left) shows the global binding orientation.

**Supplementary Figure 17**: Synthetic mass spectrometry for targeting PSMA aptamers

**Supplementary Figure 18**: Fluorescence microscopy imaging showing the Cy3-labeled PSMA-targeting aptamers conjugated to the cell membrane surface via click chemistry (Scale bar, 50 μm).

**Supplementary Figure 19:** Validation of azide retention and physiological stability of pc-CMNPs. (A) Confocal laser scanning microscopy images confirming successful PSMA aptamer conjugation and intact azide functionality after nanocarrier preparation. Cell membranes metabolically labeled with Ac4ManNAz were subjected to repeated freeze–thaw lysis and membrane extrusion to generate ~200 nm azide-presenting nanocarriers. The nanoparticles were subsequently incubated with a PSMA aptamer bearing a 5′ DBCO and a 3′ Cy3 moiety (PSMA-apt-Cy3), followed by counterstaining with the lipophilic membrane dye DiO. Colocalization (yellow) of Cy3 (red) and DiO (green) signals in the merged image demonstrates that azide groups survived the freeze–thaw and extrusion processes, permitting efficient copper-free click-chemistry-mediated aptamer anchoring (Scale bar, 2 μm). (B, C) Colloidal stability of pc-CMNPs under physiological conditions. pc-CMNPs were incubated in cell culture medium supplemented with 10% FBS, and their (B) size and (C) polydispersity index (PDI) were monitored by dynamic light scattering at the indicated time points over 72 h. Data are presented as mean ± SD (n = 3).

**Supplementary Figure 20:** Optimization of N/P ratio for D1-plasmid encapsulation by CMNPs. Agarose gel electrophoresis retardation assay showing the encapsulation efficiency of D1-plasmid at varying N/P ratios. Free D1-Plasmid (D1) migrated as a distinct band, whereas D1@CMNPs-1 (N/P = 1.2) and D1@CMNPs-2 (N/P = 2.4) exhibited partial retardation, indicating incomplete plasmid condensation. At N/P = 4.7 (D1@CMNPs-3), the plasmid band was fully retained in the loading well, demonstrating near-complete encapsulation (>90%). Addition of Triton X-100 to D1@CMNPs-3 resulted in band recovery, confirming that the absence of migration was due to effective entrapment rather than plasmid degradation. Based on these results, an N/P ratio of 4.7 was selected for subsequent pc-CMNP preparations to ensure robust plasmid loading while avoiding the elevated cytotoxicity associated with excessive cationic charge (N/P > 5).

**Supplementary Figure 21:** Effect of D1@pc-CMNP administration dose on transfection efficiency, GPX4 Degradation, and cell viability. **(A)** Western blot analysis of GPX4 protein levels in 22RV1 cells treated with different pc-CMNP formulations per 5×103 cells (a: Control; b: 0.005μg D1 plasmid in 0.2μg pc-CMNP, c: 0.010μg D1 plasmid in 0.2μg pc-CMNP; d: 0.015μg D1 plasmid in 0.2μg pc-CMNP; e: 0.025μg D1 plasmid in 0.2μg pc-CMNP; f: 0.050μg D1 plasmid in 0.4μg pc-CMNP). **(B)** Heatmap summarizing transfection efficiency, GPX4 protein expression, and cell viability across formulations a-f. **(C)** Representative fluorescence microscopy images showing GFP expression (green) and merged (bright-field and GFP) views for each formulation, indicating transfection efficiency (Scale bar, 100 μm).

**Supplementary Figure 22:** Semi-quantitative analysis of ferroptosis-related protein expression in 22RV1 **(A)** and PC3 **(B)** cells. Detection of malondialdehyde (MDA) levels in 22RV1 **(C)** and PC3 **(D)** cells treated with different formulations. Data are presented as MDA content (nmol/mg protein).
